# Synaptotagmin-13 orchestrates pancreatic endocrine cell egression and islet morphogenesis

**DOI:** 10.1038/s41467-022-31862-8

**Published:** 2022-08-04

**Authors:** Mostafa Bakhti, Aimée Bastidas-Ponce, Sophie Tritschler, Oliver Czarnecki, Marta Tarquis-Medina, Eva Nedvedova, Jessica Jaki, Stefanie J. Willmann, Katharina Scheibner, Perla Cota, Ciro Salinno, Karsten Boldt, Nicola Horn, Marius Ueffing, Ingo Burtscher, Fabian J. Theis, Ünal Coskun, Heiko Lickert

**Affiliations:** 1 Institute of Diabetes and Regeneration Research, Helmholtz Zentrum München, Neuherberg, Germany; 2 German Center for Diabetes Research (DZD), Neuherberg, Germany; 3 Institute of Computational Biology, Helmholtz Zentrum München, Neuherberg, Germany; 4 Technical University of Munich, School of Life Sciences Weihenstephan, Freising, Germany; 5 Technische Universität München, School of Medicine, München, Germany; 6 Paul Langerhans Institute Dresden of the Helmholtz Zentrum Munich at the University Clinic Carl Gustav Carus, TU Dresden, Dresden, Germany; 7 Institute for Ophthalmic Research, Center for Ophthalmology, University of Tübingen, Tübingen, Germany; 8 Technical University of Munich, Department of Mathematics, Garching b, Munich, Germany; 9 Center of Membrane Biochemistry and Lipid Research, Carl Gustav Carus School of Medicine, Technische Universität Dresden, Dresden, Germany; 10 Present Address: SOTIO a.s, Jankovcova 1518/2, Prague, Czech Republic

**Keywords:** Cell polarity, Morphogenesis, Microtubules, Endocrinology, Endocytosis

## Abstract

During pancreas development endocrine cells leave the ductal epithelium to form the islets of Langerhans, but the morphogenetic mechanisms are incompletely understood. Here, we identify the Ca^2+^-independent atypical Synaptotagmin-13 (Syt13) as a key regulator of endocrine cell egression and islet formation. We detect specific upregulation of the *Syt13* gene and encoded protein in endocrine precursors and the respective lineage during islet formation. The Syt13 protein is localized to the apical membrane of endocrine precursors and to the front domain of egressing endocrine cells, marking a previously unidentified apical-basal to front-rear repolarization during endocrine precursor cell egression. Knockout of Syt13 impairs endocrine cell egression and skews the α-to-β-cell ratio. Mechanistically, Syt13 is a vesicle trafficking protein, transported via the microtubule cytoskeleton, and interacts with phosphatidylinositol phospholipids for polarized localization. By internalizing a subset of plasma membrane proteins at the front domain, including α6β4 integrins, Syt13 modulates cell-matrix adhesion and allows efficient endocrine cell egression. Altogether, these findings uncover an unexpected role for Syt13 as a morphogenetic driver of endocrinogenesis and islet formation.

## Abstract (web summary)

How pancreatic islets of Langerhans are built during development is incompletely understood. Here the authors find that Synaptotagmin-13 mediates remodeling of cell-matrix adhesion to regulate endocrine cell egression and islet morphogenesis.

## Introduction

**Fig. 1:**
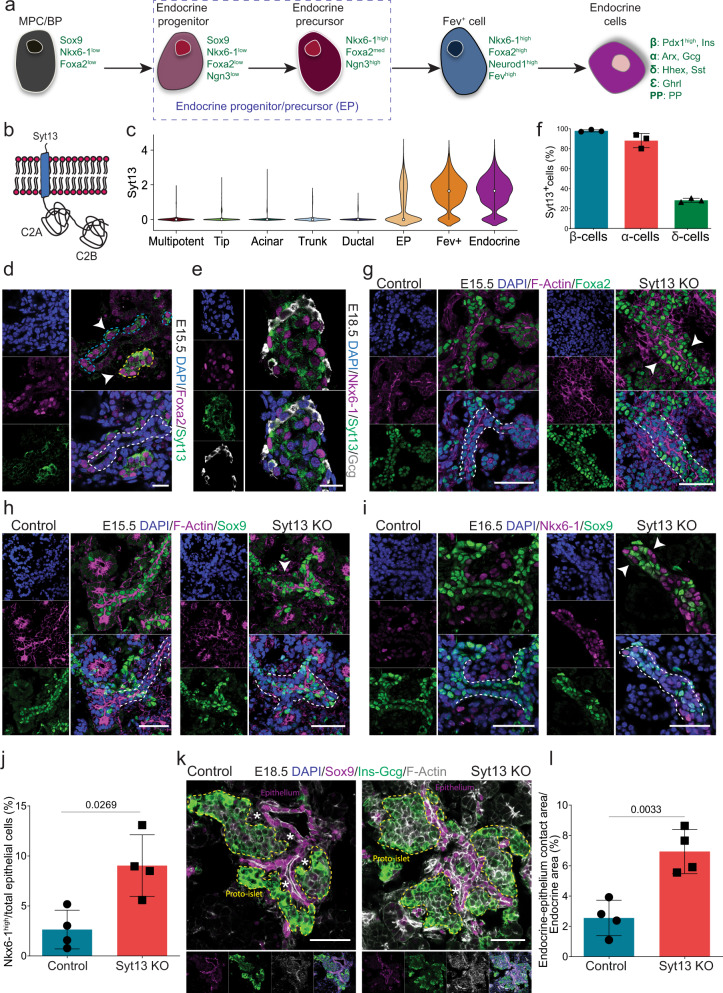
Loss of Syt13 hampers endocrine cell egression. **a** Schematic picture of stepwise endocrine lineage formation. MPC, multipotent progenitor cells; BP bipotent progenitors. **b** Schematic representation of Syt13 protein. **a** and **b** are created by the authors. **c** Violin plot showing Syt13 expression in pancreatic lineages in scRNA-seq data of mouse endocrinogenesis (E12.5-E15.5). **d** Syt13 expression in pancreatic epithelial cells (Foxa2^high^, arrowhead). **e** Expression of Syt13 in β cells (Nkx6-1^high^) and α cells (Glucagon^+^). **f** The percentage of β, α and δ cell expressing Syt13 in pancreatic sections at E18.5. *n* = 3 independent pancreata for **e** and **f**. **g** A multi-layer epithelium (arrowheads) appears in Syt13 KO pancreata. The extra layer is composed of Foxa2^high^ cells. **h**, **i** The cells in the extra epithelial layer in Syt13 KO pancreata are negative for Sox9 (arrowhead in **h**) but express high levels of Nkx6-1 (arrowheads in **i**). **j** Quantification of the number of Nkx6-1^high^/Sox9^-^ cells within or associated with the epithelium. *n* = 4 independent pancreata for **i** and **j**. **k** IHC analysis of the proto-islet position to the epithelium. * shows the area where proto-islets are not in direct contact with the nearby epithelium. **l** Quantification of contact area between proto-islets and the epithelium in Syt13 KO and control mice at E18.5. *n* = 4 independent pancreata for **k** and **l**. White dashed lines indicate epithelium. Blue dashed lines indicate newly formed endocrine cells. Yellow dashed lines indicate proto-islet. Scale bar 20 µm **d**; 50 µm (**e**, **g**–**i**, **k**). Representative pictures from three independent experiments (**d**, **g**, **h**). All statistics have been done using two-sided *t*-test. Data are represented as mean ± SD. Source data are provided as a Source Data file.

The pancreatic endocrine cells (α, β, δ, Ɛ, and PP cells) regulate glucose homeostasis. How endocrine lineage segregation is interlinked with morphogenetic programs is not well understood. During endocrinogenesis a subset of multi-/bipotent cells in the epithelium gradually express the transcription factor (TF) Neurogenin 3 (Neurog3; hereafter called Ngn3) to sequentially generate endocrine progenitors (Ngn3^low^) and precursors (Ngn3^high^)^1^. The Ngn3^high^ precursors give rise to different endocrine cell types via a transient intermediate cell population expressing the TF fifth ewing variant protein (Fev)^2^ (Fig. 1a). During stepwise lineage differentiation and morphogenesis endocrine cells egress from the ductal epithelium (also known as endocrine cell delamination) and cluster into proto-islets^3–5^. After induction of Ngn3 endocrine progenitors/precursors (EPs) reduce the apical plasma membrane (PM) area to form a tether structure, which ultimately undergoes abscission^6–9^. This sequence of events is controlled by cytoskeletal dynamics and small GTPases, such as Cdc42 and Rac1, that coordinate local endocrine cell egression to form proto-islets described as the peninsular model^10–12^. However, the possible contribution of asymmetric cell division^4,13^, endocrine cell migration^14,15^, and epithelial-to-mesenchymal transition (EMT)^8,16^ to endocrine cell egression is still a matter of debate. Furthermore, it has yet to be identified which morphogenetic drivers coordinate cell dynamics during this process. Further, the molecular mechanisms that compel egressing cells to push or breakthrough the basal lamina are unknown. Deciphering these mechanisms will help to develop tissue engineering strategies for islet cell-replacement therapy as well as identify new targets for preventing epithelial cell dissemination during cancer metastasis.

Here, we report Synaptotagmin-13 (Syt13) (Fig. 1b) as a major regulator of endocrine cell egression and islet morphogenesis during endocrinogenesis. Syt13 is an atypical member of the Synaptotagmin family of membrane trafficking proteins, which are known to be involved in intracellular vesicle trafficking and exocytosis. This protein family consists of 17 members and each possesses a transmembrane sequence connected to two lipid-binding cytoplasmic domains (C2A and C2B), responsible for docking and fusion of the carrier vesicles to the target membrane^17,18^. Compared to well-known members such as Syt1 and Syt2, Syt13 does not have an N-terminal sequence preceding the transmembrane region and does not interact with membranes in a calcium (Ca^2+^)-dependent manner^19,20^. Despite the involvement of classical Synaptotagmins proteins in vesicle exocytosis, the cellular and molecular functions of atypical members, such as Syt13, are less described.

By combining mouse knock-out studies, high-resolution imaging, cell biological assays, and single-cell mRNA profiling, we identified a critical function of Syt13 in endocrine cell morphogenesis during pancreas development. We observed a switch from apical-basal to front-rear polarization during endocrine cell egression. Importantly, Syt13 accumulates at the front domain of egressing endocrine cells and modulates cell-matrix adhesion. Altogether, these findings implicate a morphogenetic role of Syt13 during islet morphogenesis that is nonredundant and unexpected in comparison to the other Synaptotagmin proteins.

## Results

### Syt13 function is required for endocrine cell egression

In a global gene expression profiling during pancreas development *Syt13* expression was previously identified at the peak of endocrinogenesis^21^. To further pinpoint the temporal and cell-type-specific expression patterns of *Syt13* mRNA, we used single-cell RNA-sequencing (scRNA-seq) data obtained from mouse pancreatic epithelial cells that were sampled from E12.5-15.5^22^. *Syt13* expression was specific to EPs and their respective lineage (Fig. 1c). Moreover, *Syt7/SYT7* and *Syt13/SYT13* were the first synaptotagmins to be expressed during mouse in vivo endocrinogenesis (Fig. S1a) and human in vitro endocrine cell differentiation^23^ (Fig. S1b). Consistent with the previous results showing that the TF Foxa2 binds to the *Syt13* gene body^21^, we identified that Syt13 protein is synthesized in cells that express high levels of Foxa2 (Foxa2^high^) either leaving the ductal epithelium (Fig. 1d, blue dashed lines) or clustered in proto-islets (Fig. 1d, yellow dashed line). Further analysis revealed that Syt13 protein expression is restricted to the endocrine lineage and that it is synthesized in a major fraction of embryonic α and β cells, but only in a minor fraction of δ cells (Fig. 1e, f). To uncover the function of Syt13 during endocrinogenesis, we generated a Syt13 knockout (KO) mouse line using a targeted genetrap mESC clone (EUCOMM), in which the critical exon 2 was removed. This strategy resulted in the generation of a Flox (F)-deleted (FD) alleles and whole-body *Syt13*^*FD/FD*^ knock-out mice (Syt13 KO) (Fig. S1c–g). We found that control and *Syt13*^*FD/FD*^ embryos were of similar size and weight at E18.5 (Fig. S1h, i). The Mendelian ratio of the heterozygous (*Syt13*^*+/FD*^) intercrosses showed no lethality of *Syt13*^*FD/FD*^ at embryonic but at early postnatal stages (Fig. S1j). The gross morphology of the control and *Syt13*^*FD/FD*^ pancreata were comparable at E18.5 (Fig. S1k). As the heterozygous mice showed similar phenotypes to the wild type (WT) animals, we refer to both genotypes hereafter as controls. We found no evident histological alterations in the *Syt13*^*FD/FD*^ pancreatic epithelium at E12.5 and E13.5 (Fig. S2a, b). Further analysis revealed no changes in apical-basal polarity of *Syt13*^*FD/FD*^ ductal epithelial and acinar cells (Fig. S2c, d). These data demonstrate that ductal and acinar differentiation and morphogenesis are normal upon *Syt13* deletion, and that Syt13 expression and function are highly specific to the endocrine lineage.

Starting from E14.5, striking alterations in epithelial organization appeared in the Syt13 KO pancreata. While the pancreatic epithelium of control mice consisted of a single-layer of Foxa2^low^ cells, the Syt13 KO pancreata contained a multi-layer epithelium. These additional layers consisted mostly of Foxa2^high^ cells (Fig. 1g, Fig. S2e) and were negative for the ductal cell marker Sox9 (Fig. 1h), indicating a defect during endocrine lineage acquisition. In support of this, we observed an increased number of retained EPs/endocrine cells (Nkx6-1^high^/Sox9^−^) within the *Syt13*^*FD/FD*^ epithelium as compared to the control (Fig. 1i, j). Moreover, we detected an increased direct attachment between proto-islets and the epithelium in *Syt13*^*FD/FD*^ compared to control pancreata (Fig. 1k, l, Fig. S3a, b, Movie S1, 2). The typical proto-islet rearrangement, in which α cells are at the periphery and β cells at the core, was also disrupted in Syt13 KO pancreata (Fig. S3c). To dissect at which cell state Syt13 regulates endocrine cell egression, we generated two lineage and cell type-specific Syt13-conditional KO (CKO) mice using *Ngn3*^*Cre/+*^ and *Ins1*^*Cre/+*^ Cre-driver lines^24,25^ to specifically delete Syt13 in EPs and β cells, respectively. We found an impairment in endocrine cell egression in *Ngn3*^*Cre/+*^; *Syt13*^*F/FD*^, but not in *Ins1*^*Cre/+*^; *Syt13*^*F/FD*^ animals (Fig. S3d, e). Hence, Syt13 function is crucial for endocrine cell egression and islet formation in Ngn3^+^ EPs, but not in newly formed Ins^+^ β cells that already left the ductal epithelium.

### High expression levels of *Syt13* prime β-cell fate

**Fig. 2:**
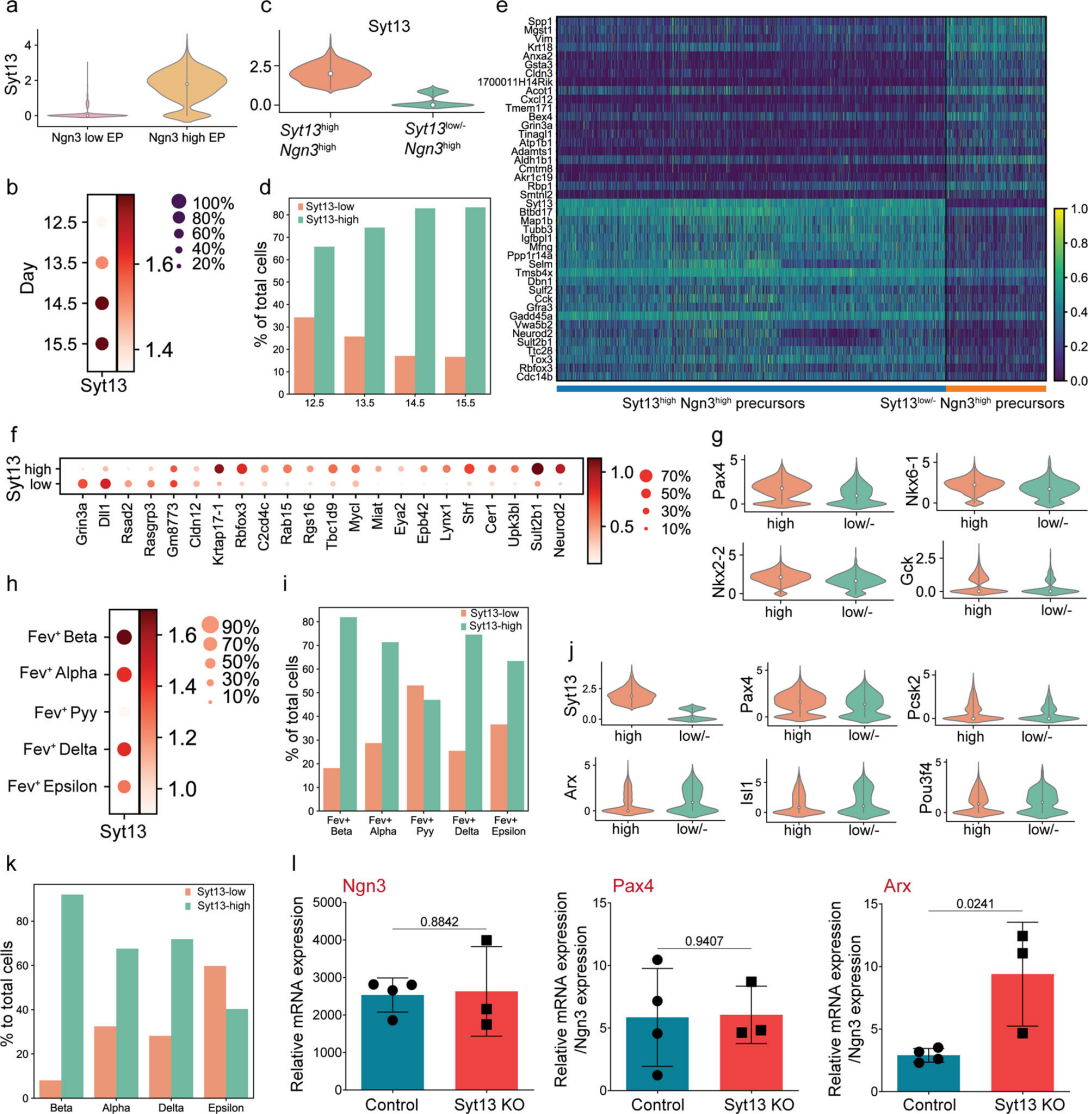
Increased expression levels of *Syt13* associate with the β cell program. **a** Violin plot of *Syt13* expression levels in endocrine progenitors and precursors. **b** Dot plot of *Syt13* expression in endocrine precursors at different developmental stages. **c** Violin plot shows the dividing of the endocrine precursors into *Syt13*^high^ and *Syt13*^low/−^ clusters. **d** The percentage of *Syt13*^high^ *Ngn3*^high^ and *Syt13*^low/−^ *Ngn3*^high^ cells at different developmental stages. **e** Heatmap of top 21 differentially expressed genes in *Syt13*^high^ and *Syt13*^low/−^ precursors. **f** Dot plot indicating the differential expression of EP-signature genes in *Syt13*^high^ and *Syt13*^low/−^ precursors. **g** Violin plots showing the expression of β cell-specific genes in *Syt13*^high^ and *Syt13*^low/−^ precursors. **h** Dot plot of *Syt13* expression in different *Fev*^+^ cells from E12.5-E15.5. **i** The percentage of *Syt13*^high^ and *Syt13*^low/−^ cells in different *Fev*^+^ cell types. **j** Violin plots showing the expression of β and α cell-specific genes in *Syt13*^high^ and *Syt13*^low/−^ *Fev*^+^ cells. **k** The percentage of *Syt13*^high^ and *Syt13*^low/−^ cells in different hormone^+^ endocrine cell types from E12.5–E15.5. **l** qPCR analysis of NVF^+^ cells sorted from pancreatic epithelium from NVF;control (WT) and NVF;Syt13 KO mice at E15.5. *n* = 4 (control) and *n* = 3 (KO) independent pancreata. All statistics have been done using two-sided *t*-test. Data are represented as mean ± SD. Source data are provided as a Source Data file.

We next dissected the expression dynamics of Syt13 in endocrine lineages using the previously published scRNA-seq data^22^. *Syt13* expression levels were high in *Ngn3*^high^ precursors, compared to very low levels in *Ngn3*^low^ progenitors (Fig. 2a). Moreover, *Ngn3*^high^ precursors from E14.5 and E15.5 had higher levels of *Syt13* than those from E12.5 and E13.5 (Fig. 2b). To investigate whether Syt13 expression levels correlate with different endocrine cell fates, we divided *Ngn3*^high^ precursors from E12.5–15.5 into two clusters expressing high levels (*Syt13*^high^) versus no/low levels of *Syt13* (*Syt13*^low/−^) (Fig. 2c). The fraction of *Syt13*^high^ cells increased from E12.5 to E15.5 (Fig. 2d). Differential gene expression analysis between *Syt13*^high^ and *Syt13*^low/−^ precursors identified several previously reported EP-signature genes^22^ (Fig. 2e, Supplementary Data 2). Most of the signature genes, likely associated with the β cell fate (e.g. *Neurod2*, *Sult2b1* and *Upk3bl*) were highly expressed in *Syt13*^high^ precursors. In contrast, genes likely associated with the α cell fate (e.g. *Dll1*, *Rsad2*, and *Rasgrp3*)^22^ were highly expressed in *Syt13*^low/−^ precursors (Fig. 2f). Furthermore, the expression levels of *Pax4*, *Gck*, *Nkx6-1*, and *Nkx2-2* were higher in *Syt13*^high^ than in *Syt13*^low/−^ precursors, suggesting that *Syt13*^high^ *Ngn3*^high^ cells are primed towards β cell fate (Fig. 2g).

We then analyzed Syt13 expression in *Fev*^+^ cells and found higher levels in *Fev*^+^β than in other *Fev*^+^ endocrine subtypes (Fig. 2h). Additionally, the proportion of *Syt13*^high^ cells in *Fev*^+^β cells was higher than that in *Fev*^+^α, *Fev*^+^δ and *Fev*^+^ε and *Fev*^+^Pyy cells (Fig. 2i). Next, we divided all *Fev*^+^ cells into *Syt13*^high^ and *Syt13*^low/−^ cells and performed differential gene expression analysis (Fig. S4a, Supplementary Data 2). *Pax4* and *Pcsk2* were upregulated in *Syt13*^high^, while *Arx*, *Isl1*, and *Pou3f4* were upregulated in *Syt13*^low/−^ *Fev*^+^ cells (Fig. 2j). These data suggest that higher expression levels of *Syt13* in *Fev*^+^ cells correlate with a β cell program. Consistent with *Syt13* expression in endocrine precursors and *Fev*^+^ cells, the fraction of *Syt13*^high^ cells was highest in β cells compared to other hormone^+^ endocrine subtypes (Fig. 2k). Moreover, partition-based graph abstraction (PAGA) analysis showed high connectivity between *Syt13*^high^ precursors, *Syt13*^high^ *Fev*^+^ cells and β cells (Fig. S4b). In line with these findings, scRNA-seq data of human fetal pancreas^26^ identified higher mRNA expression of *SYT13* in β cells and their precursors than in α cells and their precursors (Fig. S4c). To support the findings from scRNA-seq results, we performed qPCR analysis of FACS isolated Ngn3^+^ cells from E15.5 *NVF*; *Syt13*^*FD/FD*^ pancreata, which were obtained by crossing *Syt13*^*+/FD*^ mice with homozygous Ngn3-Venus fusion (NVF) reporter animals^22^. We found comparable levels of *Ngn3* in the control and *Syt13*^*FD/FD*^ Ngn3^+^ cells. Furthermore, an increased expression level of *Arx*, but not *Pax4*, was observed in the *Syt13*^*FD/FD*^ Ngn3^+^ cells compared to the controls (Fig. 2l). Collectively, these analyses demonstrate that high physiological levels of *Syt13* mRNA expression in endocrine precursors and *Fev*^+^ cells correlate with β cell fate acquisition.

### Lack of Syt13 reduces β-cell specification

**Fig. 3:**
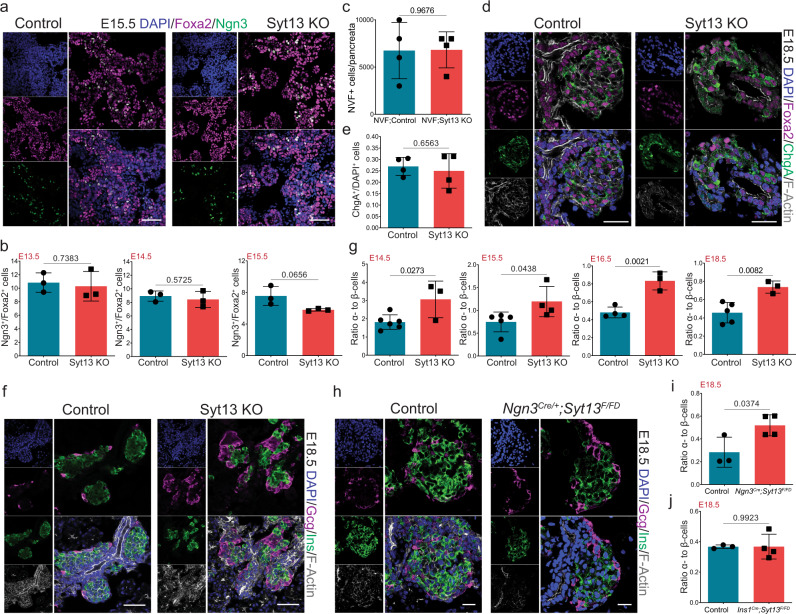
Loss of Syt13 reduces β-cell specification. **a** IHC analysis showing Ngn3^+^ cells in control and Syt13 KO mice. **b** Quantification of the percentage of Ngn3^+^ cells. *n* = 3 independent pancreata for **a** and **b**. **c** Total number of NVF (Ngn3^+^) cells in E15.5 pancreata sorted by FACS. *n* = 4 independent pancreata. **d** IHC analysis of ChgA in pancreatic sections. **e** Quantification of ChgA^+^ cells in E18.5 pancreata. *n* = 4 independent pancreata for **d** and **e**. **f** IHC analysis of pancreatic sections from control and Syt13 KO mice. **g** Quantification of the α/β cells ratio in pancreatic sections from control and Syt13 KO mice. *n* = 6 controls and 3 KO (E14.5), 5 controls and 4 KO (E15.5), 4 controls and 3 KO (E16.5), and 5 controls and 3 KO (E18.5) independent pancreata for **f** and **g**. **h** IHC analysis of pancreatic sections from control and *Ngn3*^*Cre/+*^*;Syt13*^*F/FD*^ mice. (**i**, **j**) Quantification of the α/β cells ratio in *Ngn3*^*Cre/+*^*;Syt13*^*F/FD*^, and *Ins1*^*Cre/+*^*;Syt13*^*F/FD*^ pancreata, respectively. *n* = 3 controls and 4 KO (E18.5) independent pancreata for **h**–**j**. Scale bar 50 µm (**a**, **d**, **f**); 20 µm (**h**). All statistics have been done using two-sided *t*-test. Data are represented as mean ± SD. Source data are provided as a Source Data file.

To validate the results from the scRNA-seq data, we examined the effects of Syt13 KO on endocrine lineage formation. We found comparable numbers of Ngn3^+^ cells from control and Syt13 KO mice at E13.5–15.5 (Fig. 3a, b). FACS analysis also revealed a similar number of Ngn3^+^ cells in *NVF*; *Syt13*^*FD/FD*^ and control pancreata at E15.5 (Fig. 3c). In addition, Syt13 KO and control pancreata contained a comparable fraction of cells expressing the pan-endocrine cell marker, Chromogranin A (ChgA) (Fig. 3d, e). Next, we quantified the number of α and β cells. Syt13 KO pancreata exhibited a significant increase in the number of α cells at the expense of β cells at E14.5–E18.5 (Fig. 3f, g). Importantly, we found that the deletion of Syt13 in EPs but not in the newly generated insulin-expressing cells resulted in an increase of the α  to β cell ratio (Fig. 3h–j, Fig. S4d). Together, these results support the finding from the in vivo scRNA-seq data and further indicate that Syt13 acts downstream of Ngn3 and functions in EPs to specify or determine β cell fate allocation. Thus, lack of Syt13 impairs proper endocrine cell egression and specification, further supporting previous findings that endocrine cell morphogenesis and differentiation are tightly linked^3,6^.

### Syt13 localizes at the front domain of egressing cells

**Fig. 4:**
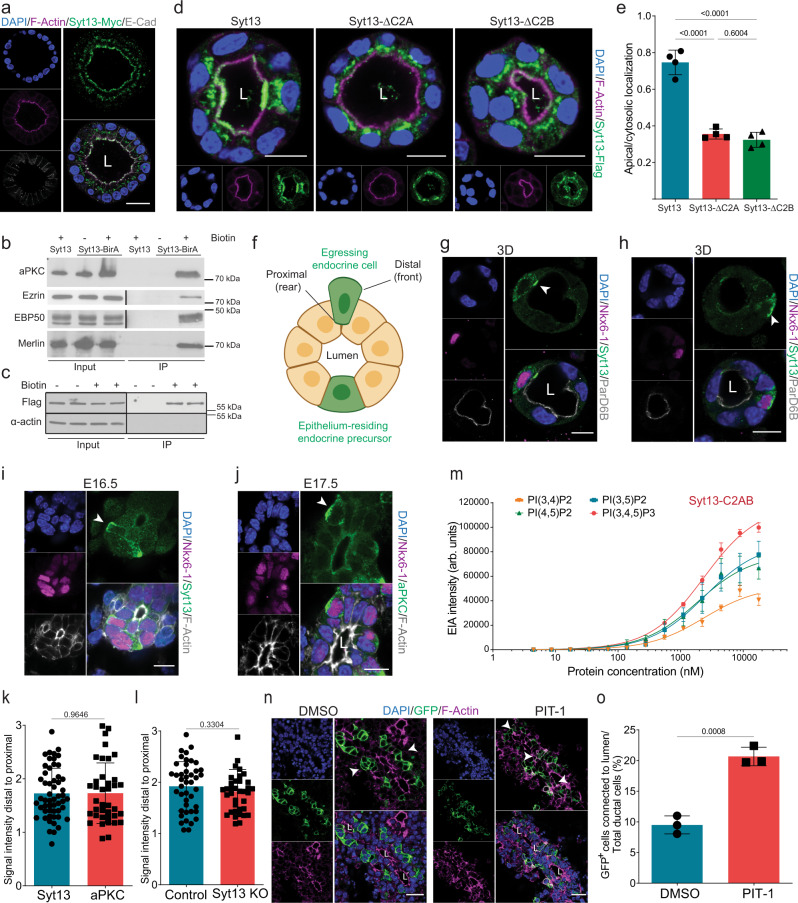
Syt13 localizes to the apical PM of epithelial and the front domain of egressing endocrine cells. **a** Syt13 localizes to the apical PM in polarized MDCK cells. **b** Western blot showing the close proximity of Syt13 with different apical proteins in MDCK cells. **c** Surface biotinylation assay showing Syt13 localization to the plasma membrane. **d** Apical localization of Syt13 full-length and the C2 truncated variants. **e** Quantification of apical localization of Syt13 protein variants. *n* = 4 independent experiments for **d** and **e**. **f** Scheme of positioning of an epithelium-residing endocrine precursor and an egressing endocrine cell. Distal (front) and proximal (rear) domains to the central lumen are defined in egressing endocrine cells. Created with BioRender.com. **g** Syt13 localizes to the apical PM (arrowhead) in differentiated endocrine cells fully residing within the pancreatic epithelium. Syt13 localizes at the front domain (arrowheads) of endocrine cells that are not in direct contact with the epithelial lumen in 3D epithelial cysts (**h**) and in vivo (**i**). **j** Pancreatic section showing the expression of aPKC at the front domain (arrowhead) of egressing endocrine cells. **k** Quantification of Syt13 and aPCK expression at the distal and proximal domains of newly formed endocrine cells. For Syt13, *n* = 54 endocrine cells from five independent pancreata (**i** and **k**). For aPKC, *n* = 38 endocrine cells from four independent pancreata (**j** and **k**). (**l**) Quantification of aPCK expression at the distal and proximal domains of newly formed endocrine cells in control and Syt13 KO pancreatic sections. For control, *n* = 43 endocrine cells from 4 independent pancreata. For KO *n* = 36 endocrine cells from three independent pancreata. **m** Binding of purified Syt13-C2AB protein variant to 100 nm sized LUVs containing different species of phosphatidylinositol phospholipids (POPC/cholesterol/phosphoinositide 65/30/5 mol %) assessed by electrochemiluminescence-based immunoassay (EIA). *n* = 3 independent experiments. **n**, **o** Treatment of explant culture of E13.5 *Ngn3*^*Cre/+*^*; ROSA26*^*mTmG/mTmG*^ pancreata with PIP3 inhibitor PIT-1 for 48 h impairs endocrine cell egression. n = 3 independent experiments. Arrowheads indicate endocrine cells. L lumen. Scale bar 10 µm (**d**, **g**–**j**); 20 µm (**a**, **n**). Representative pictures from 2 (**b**, **c**) and 3 (**a**, **g**, **h**) independent experiments. Tukey’s multiple comparisons test (**e**) and two-sided *t*-test (**k**, **l**, **o**) were used for statistics. Data are represented as mean ± SD. Source data are provided as a Source Data file.

To decipher the cellular processes by which Syt13 coordinates endocrine cell egression and islet morphogenesis, we first explored its subcellular localization. The expression of *Syt13* mRNA emerges in epithelium-residing EPs (Fig. 1c). Therefore, we first assessed the localization of the protein in Madin-Darby Canine Kidney (MDCK) cells overexpressing Syt13 (Syt13^OVE^) cultured in 3D as a model for an apical-basal polarized epithelium. Remarkably, Syt13 was specifically localized at the apical domain of polarized epithelial cysts (Fig. 4a). Proximity-dependent biotin identification (BioID) further identified the close proximity of Syt13 with several apical polarity determinants, including aPKC, Ezrin, EBP50, and Merlin (Fig. 4b). Surface biotinylation indicated the incorporation of Syt13 within the PM (Fig. 4c). A structure-function study unveiled that Syt13 requires both C2A and C2B domains for PM integration and localization (Fig. 4d, e, Fig. S5a). This PM localization differs from that of Syt1 and Syt2, which are predominantly enriched in synaptic vesicles and mediate exocytosis, suggesting a distinct cellular function of Syt13.

Next, we explored the subcellular localization of Syt13 during endocrinogenesis. Using our previously established in vitro 3D endocrine differentiation system^27^, we observed that in differentiated endocrine cells, which were still fully integrated within the pancreatic epithelium, Syt13 was detected at the apical PM domain (Fig. 4f, g, Fig. S5b). In contrast, in endocrine cells that were not directly in contact with the epithelial lumen, Syt13 was mostly found at the distal domain to the lumen (the front domain of endocrine cells) (Fig. 4f, h, i, k). This differential expression pattern of Syt13 suggests that egressing endocrine cells undergo a repolarization event. This process occurs in cells leaving an epithelium and involves remodeling of cell-cell and cell-matrix adhesion and relocating the apical determinants, Golgi apparatus, and microtubule-organizing centers (MTOCs) from the apical domain of epithelial cells to the front domain of evading cells^28^. In support of this, we also found prominent expression of the atypical protein kinase C (aPKC) at the front area of egressing cells (Fig. 4j, k, Fig. S5c) and that Syt13 and aPKC were colocalized to this domain (Fig. S5d). Next, we examined whether Syt13 acts as an upstream regulator of endocrine cell repolarization. Both control and Syt13 KO cells showed normal distribution of aPKC at the front domain of egressing cells (Fig. 4l, Fig. S5e). Furthermore, we observed similar E-Cadherin (E-Cad) localization patterns in control and Syt13 KO egressing cells (Fig. S5f) as previously reported^27^. Taken together, these data demonstrate that Syt13 is not a major regulator of endocrine cell repolarization.

We then sought to understand how Syt13 is localized to the apical membrane and the front domain. The interaction of some Synaptotagmin members such as Syt1 and Syt7 with phosphoinositide phospholipids has been shown to guide vesicles carrying these proteins to the target membranes^29,30^. Because these phospholipids are differentially localized to the apical, basolateral, front, and rear domains^28^, we investigated their direct interaction with Syt13. We conducted an in vitro lipid-binding analysis of purified recombinant Syt13 protein variants (Fig. S6a, Supplementary Data 1) to large unilamellar vesicles (LUVs). The C2A domain showed low binding property, while the C2B and C2AB domains exhibited a preference for PI(3,4,5)P3 (PIP3), PI(3,5)P2, and PI(4,5)P2, and with lower degree for PI(3,4)P2 (Fig. 4m, Fig. S6b–d). According to these data, Syt13 directly interacts with PIP3 and PIP2 phosphoinositides primarily through its C2B cytoplasmic domain. As PIP3 is enriched at the cell front domain^28^, we next assessed if inhibition of PIP3 formation or function mimics the Syt13 action during endocrine cell egression. To test this, we used explant cultures from the lineage-tracing *Ngn3*^*Cre/+*^*; ROSA26*^*mTmG/mTmG*^ pancreata (epithelial cells express Tomato and endocrine cells express GFP) and treated them with either PIP3/protein binding inhibitor (PIT-1) or the PI3K inhibitor (LY294002). Both inhibitors impaired endocrine cell egression (Fig. 4n, o, Fig. S6e, f). Next, we tested if Syt13 is also involved in endocrine lineage dynamics using a 2D culture of primary pancreatic cells (Fig. S7a, b). We crossed *Ngn3*^*Cre/+*^*; ROSA26*^*mTmG/mTmG*^ mice with *Syt13*^*FD/FD*^ line to generate the lineage-tracing Syt13 KO animals and cultured the primary pancreatic cells from these mice in the 2D system. Time-lapse imaging followed by image analysis revealed that lack of Syt13 did not alter endocrine cell morphodynamics features such as cell area, solidity, and speed of cell movement compared to control cells (Fig. S7c–e, Movie S3, 4).

### The intracellular trafficking of Syt13 requires microtubules

**Fig. 5:**
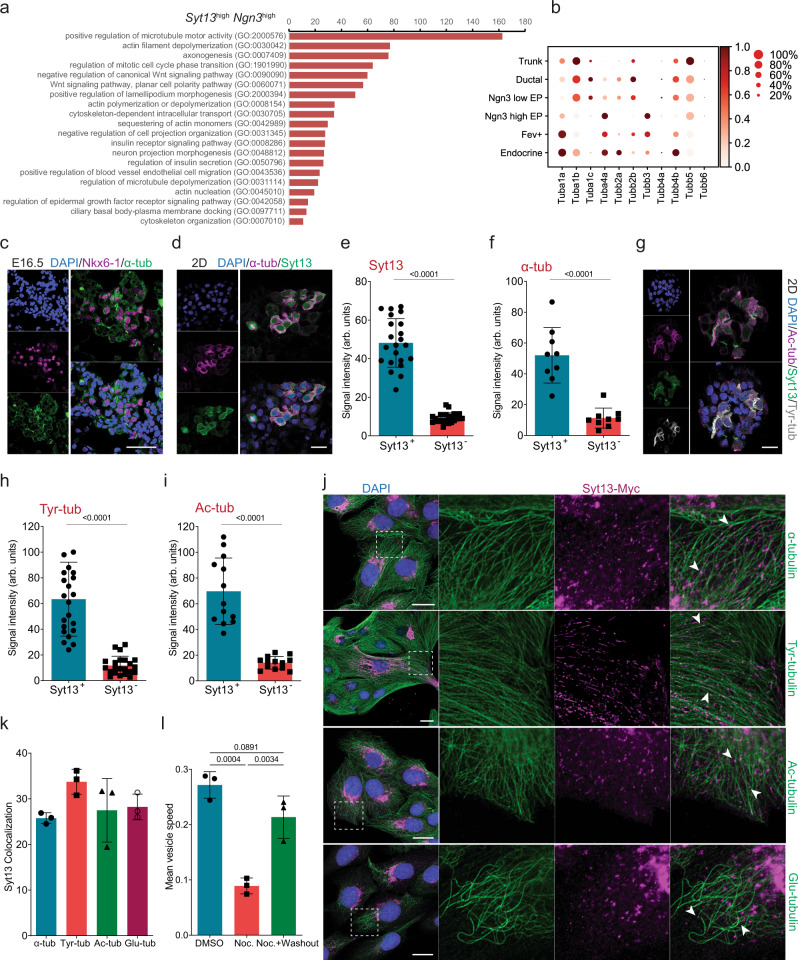
Syt13 associates with MT cytoskeleton and its intracellular trafficking relies on MT network. **a** Selected terms from the pathway analysis of top 500 upregulated genes in *Syt13*^high^ compared to *Syt13*^low/-^ precursors. **b** Dot plot of the expression levels of genes encoding different tubulin isotypes during mouse endocrinogenesis. Ngn3 low EP is endocrine progenitor and Ngn3 high EP is endocrine precursor. **c** The expression of α-tubulin in endocrine lineages. **d** IF analysis showing co-expression of Syt13 with α-tubulin in endocrine lineage in the 2D culture system. **e**, **f** Quantification of Syt13 and α-tubulin levels in Syt13-positive and Syt13-negative pancreatic cells. For Syt13, *n* = 22 cell clusters from 4 independent pancreata. For α-tubulin, *n* = 9 cell clusters from 2 independent pancreata. **g** Co-expression of Syt13 with Tyr-tub and Ac-tub in pancreatic cells in the 2D culture. **h**, **i** Quantification of Tyr-tub and Ac-tub levels in Syt13-positive and Syt13-negative pancreatic cells. For Tyr-tub, *n* = 22 cell clusters from four independent pancreata. For Ac-tub, *n* = 13 cell clusters from two independent pancreata. **j** IF analysis of Syt13 colocalization with different forms of α-tubulin in MDCK cells (arrowheads). **k** Quantification of Syt13 colocalization with different α-tubulin forms in MDCK cells. *n* = 3 independent experiments for **j**, **k**. **l** Quantification of the movement of intracellular vesicles harboring Syt13 in MDCK upon treatment with DMSO, nocodazole, and nocodazole + washout. *n* = 3 independent experiments. Scale bar 40 µm (**c**); 20 µm (**d**, **g**, **j**). Representative pictures from two independent experiments (**c**, **d**, **g**). Tukey’s multiple comparisons test (**l**) and two-sided t-test (**e**, **f**, **h**, **i**) were used for statistics. Data are represented as mean ± SD. Source data are provided as a Source Data file.

To identify the molecular components linked to Syt13 functions during endocrine cell egression, we performed pathway enrichment analysis of differentially expressed genes between *Syt13*^high^ and *Syt13*^low/−^ endocrine precursors. We found that increased *Syt13* expression in these cells was linked to differential expression of genes coding for microtubule (MT) cytoskeletal components and regulators as well as intracellular transport pathways (Fig. 5a and Supplementary Data 2). Among them, the transcript levels of *Tuba1a*, *Tuba4a*, and *Tubb3* increased during endocrinogenesis, while the levels of genes encoding other tubulin isotypes decreased or remained unchanged (Fig. 5b). Notably, mRNA levels of *Tuba1a*, *Tuba4a*, and *Tubb3* were higher expressed in *Syt13*^high^ than in *Syt13*^low/−^ precursors (Fig. S8a). Furthermore, we found increased protein levels of α-tubulin and β3-tubulin in endocrine cells and Syt13-expressing cells (Fig. 5c–f, Fig. S8b, c). α-tubulin is synthesized with a tyrosine residue at the C-terminus (Tyr-tub) and can be post-translationally modified by detyrosination, acetylation (Ac-tub), and glutamylation (Glu-tub). These post-translational modifications (PTMs) of tubulin influence the stability and dynamics of the MT cytoskeleton^31^. Using the scRNA-seq data, we evaluated expression levels of the enzymes involved in tubulin PTM, indicating increased transcripts of *α-tubulin N-acetyltransferase 1 (Atat1)* and *tubulin polyglutamylase (Ttll7)* in *Ngn3*^high^ precursors. In contrast, mRNA levels of *Svbp*, which is involved in α-tubulin detyrosination, were reduced (Fig. S8d). In agreement with the gene expression data, we also detected a significant increase in Tyr-tub and Ac-tub protein content in Syt13-expressing cells (Fig. 5g–i).

To further test the association between Syt13 and the MT cytoskeleton, we analyzed colocalization of this protein with different forms of α-tubulin in MDCK cells. Syt13 exhibited high colocalization with α-tub, Tyr-tub, Ac-tub, and Glu-tub (Fig. 5j, k). Next, we assessed the functional interactions between Syt13 and MT cytoskeleton. Time-lapse imaging of MDCK cells expressing Syt13-Venus fusion protein showed the presence of highly dynamic Syt13-positive endosomal compartments. Treatment of cells with nocodazole, a MT destabilizer, reduced the number and movement of the endosomal vesicles harboring Syt13. Importantly, washout of nocodazole restored Syt13-containing endosomal compartments and their movement (Fig. 5l, Fig. S8e–g, Movie S5). These findings indicate that the transport of Syt13-harboring vesicles occurs along MTs that likely directs and enriches this protein to the apical and front domains of endocrine precursors and the egressing cells, respectively.

### Syt13 remodels cell-matrix adhesion of egressing cells

**Fig. 6:**
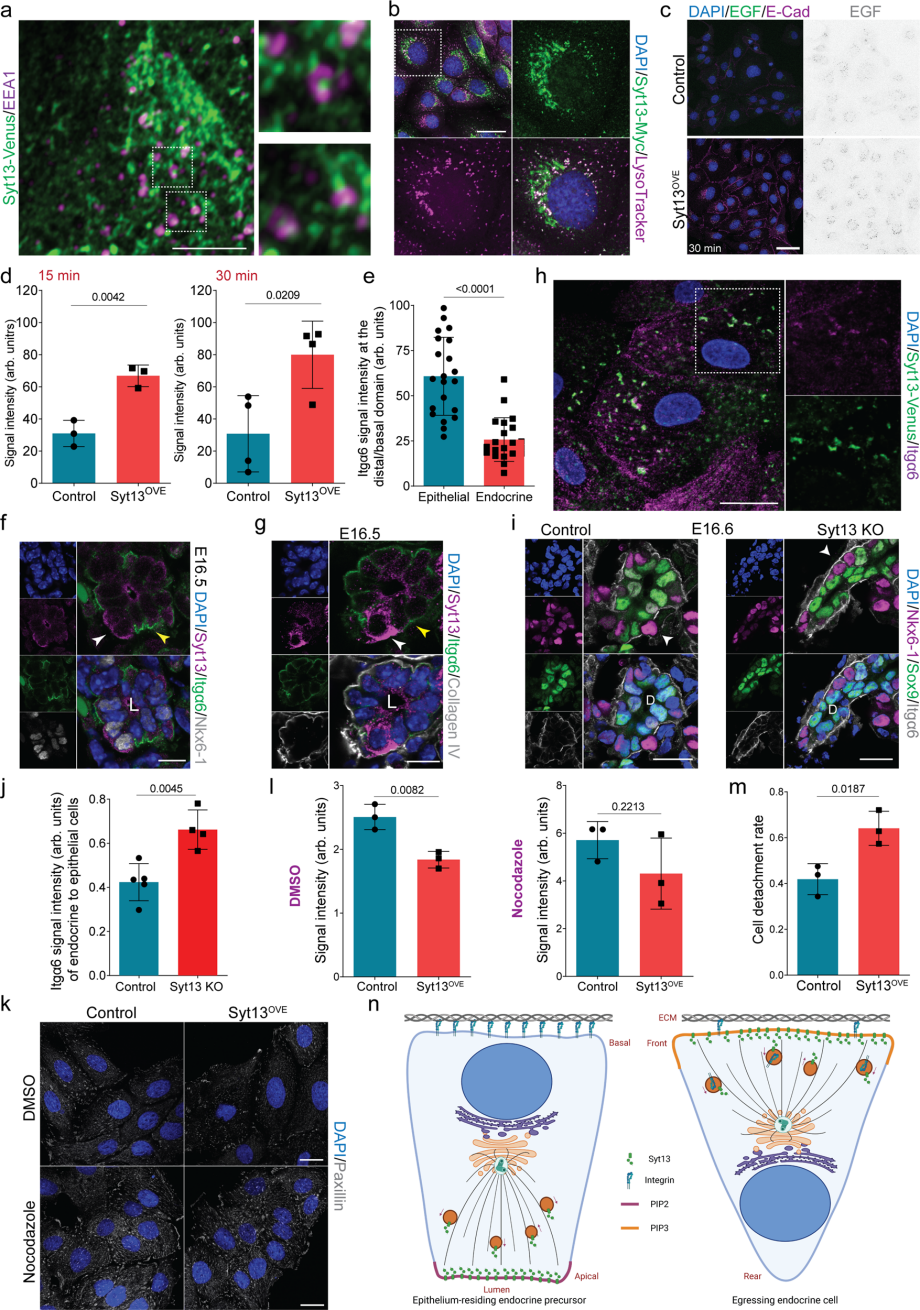
Syt13 is a trafficking protein and is involved in the remodeling of cell-matrix adhesion. **a** Colocalization of Syt13 with EEA1 puncta in MDCK cells. **b** Colocalization of Syt13 with LysoTracker in MDCK cells. **c**, **d** IF and quantification of EGF uptake in control and Syt13^OVE^ MDCK cells. *n* = 3 (15 min) and *n* = 4 (30 min) independent experiments. **e** Quantification of Itgα6 levels at the basal/distal domain of embryonic epithelial and endocrine cells in 3D culture. *n* = 21 cyst structures cultured in 3D from three independent pancreata. **f**, **g** Reduction of Itgα6 levels in the distal domain of endocrine cells (white arrowheads) coincides with the expression of Syt13 at this domain. Yellow arrowheads show higher levels of Itgα6 at the basal domain of neighboring epithelial cells. **h** Colocalization of Itgα6 with Syt13-positive vesicles in MDCK cells. **i** IHC analysis shows retention of Itgα6 at the distal domain (arrowheads) of Syt13 KO endocrine cells. **j** Quantification of Itgα6 at the distal domain of endocrine cells compared to the basal domain of nearby epithelial cells. *n* = 5 control and 4 KO independent pancreata for **i** and **j**. **k**, **l** Immunostaining and quantification of paxillin in control and Syt13^OVE^ MDCK cells treated with DMSO or 10 μM nocodazole. *n* = 3 independent experiments. **m** Quantification of cell detachment rate in control and Syt13^OVE^ MDCK cells. *n* = 3 independent experiments. **n** Scheme of the model of Syt13 function during endocrine cell egression. Created with BioRender.com. Scale bar 5 µm (**a**); 10 µm (**f**, **g**); 20 µm (**h**, **i**, **k**); 50 µm (**b**, **c**). Representative pictures from 3 (**a**, **b**) and 2 (**h**, **f**, **g**) independent experiments. All statistics have been done using two-sided *t*-test. Data are represented as mean ± SD. Source data are provided as a Source Data file.

We next investigated whether and how Syt13 vesicle trafficking function regulates endocrine cell egression. We conducted BioID proximity labeling in MDCK cells followed by mass spectrometry analysis to find proteins in close vicinity to Syt13 (Supplementary Data 3). Pathway analysis revealed protein interactions with the cytoskeleton, motor proteins, vesicle trafficking, internalization and degradation machinery, and cell-cell and cell-matrix adhesions (Fig. S9a). Close proximity of several proteins involved in vesicle trafficking including Caveolin-1 (Cav-1), hepatocyte growth factor-regulated tyrosine kinase substrate (Hgs or Hrs), and Small GTPases Rab5, Rab7, and Rab11 was confirmed using co-IP experiments (Fig. S9b). Following that, we analyzed the colocalization of Syt13 with the endomembrane system in Syt13^OVE^ MDCK cells. We found enrichment of Syt13 in the Golgi apparatus (Fig. S9c) and its colocalization with the early endosomal marker EEA1 (early endosome antigen 1) (Fig. 6a). Moreover, we observed high colocalization of Syt13 with lysosomal compartments positive for LysoTracker (69.8 ± 10.5%) and lysosome-associated membrane protein‑1 (LAMP1) (Fig. 6b, Fig. S9d, Movie S6). To examine the endocytic function of Syt13, we performed endocytosis assays using Transferrin (Tf) and EGF as ligands. Syt13 gain-of-function in MDCK cells increased the levels of EGF internalization (Fig. 6c, d, Fig. S9e), but did not alter the rate of Tf uptake (Fig. S9f, g), indicating the involvement of Syt13 in the internalization of a subset of plasma membrane proteins.

We then asked what Syt13 internalizes at the front domain for endocrine cell egression. One possible scenario is that the endocytic function of Syt13 could contribute to the removal of the cell-matrix junctions. Since such structures are enriched for integrins, we first determined which integrin subunits are expressed during endocrinogenesis. Analysis of mouse scRNA-seq data revealed different expression levels of *Itga3, Itga5, Itga6, Itga9, Itgav, Itgb1, Itgb4, Itgb5* and *Itgb6* genes in epithelial cells (Fig. S9h). Most of these integrin subunits were downregulated at EP and *Fev*^+^ states (Fig. S9h). Among the expressed subunits, an increased expression of α6 integrin (Itgα6) in acinar cells compared to lower levels in ductal and endocrine lineages has been previously reported^32^. We detected a further reduction in the levels of Itgα6 in endocrine clusters compared to ductal epithelial cells (Fig. S9i). However, we found similar laminin expression surrounding endocrine cell clusters and epithelial cells (Fig. S9i). Notably, the levels of Itgα6 at the distal domain of the endocrine cells residing within the epithelium were lower than the basal domain of the nearby epithelial cells (Fig. 6e). This indicates that the onset of Itgα6 reduction precedes the egression process. The reduction in Itgα6 levels at the front domain of endocrine cells was accompanied by Syt13 enrichment at this domain (Fig. 6f, g), and Itgα6 was found to colocalizes with Syt13-carrying vesicles (Fig. 6h). Importantly, we detected increased levels of Itgα6 at the front domain of Syt13 KO endocrine cells compared to control cells (Fig. 6i, j). Similar increased levels were also observed for Itgβ4 subunit (Fig. S9j). Finally, we tested if Syt13 modulates cell-matrix adhesion. Syt13^OVE^ MDCK cells treated with DMSO but not nocodazole exhibited reduced focal adhesion (FA) area, marked by the FA-associated adapter protein paxillin, in vitro (Fig. 6k, l). Moreover, by performing a cell detachment assay, we found reduced cell-matrix adhesion upon Syt13 gain-of-function in MDCK cells (Fig. 6m, Fig. S9k). Together, these data suggest an important role for Syt13 in regulating cell-matrix adhesion at the front domain of egressing endocrine cells to leave the ductal epithelium.

## Discussion

Here we revisited the cellular and molecular aspects of pancreatic endocrine cell egression during endocrinogenesis. Setting our findings in context of previous studies, we propose a model in which, endocrine cell egression initiates at EP cell state^8^. The onset of Ngn3 expression in EPs triggers the expression of morphogenetic regulators, i.e. cytoskeletal components, MT modulators, and the vesicle trafficking protein Syt13. First, endocrine precursors condense their apical PM^6^ marked by Syt13, reduce their apical-basal polarity and gradually remodel tight and adherens junctions^27^. Then, Syt13 relocates to the front domain of the egressing cells, indicating a switch from apical-basal to front-rear polarity. At the rear part of the egressing cells, the apical plasma membrane domain further narrows, resulting in the formation of a tether structure connecting the cells with the ductal epithelial sheet^7^. At the front domain Syt13 modulates cell-matrix adhesion that together with the abscission of the tether structure at the rear end^7^ enforce the detachment of the egressing cells from the epithelium (Fig. 6n).

Currently, the cellular and molecular processes that coordinate endocrine cell egression are ill defined. We describe a previously unknown cell repolarization process during endocrine cell egression. Prior studies have shown reduction of the apical domain that ultimately leads to loss of apical-basal polarity in endocrine cells^6,27^. Here, we provide evidence that after loss of apical-basal polarity, egressing and likely migrating endocrine cells acquire a front-rear polarity. Importantly, we found Syt13 as a marker protein for this repolarization event. However, our data suggest that Syt13 is not a key regulator of endocrine cell repolarization, but acts as an effector protein to modulate the front domain and cell-matrix interactions. Intracellular vesicle trafficking and the cytoskeletal network likely coordinate this repolarization process. This notion is supported by the increased levels of MTs and their PTMs in cells expressing Syt13 and the dependency of Syt13 intracellular movement to the MT cytoskeleton.

Syt13 exhibited direct interaction with phosphoinositide phospholipids. The interaction with PIP3 likely orients vesicles carrying Syt13 to the front domain of egressing endocrine cells. Since PIP3 is accumulated at the leading edge of migratory cells^33^, it is tempting to speculate that Syt13 also belongs to this dynamic membrane domain. The pharmacological inhibition of PIP3 and phosphoinositide 3-kinases also reduced endocrine cell egression, suggesting a functional interlink between PI3K signaling and Syt13. This view is supported by the involvement of PI3K pathway in regulating endocrine progenitor apical domain narrowing^6^, as well as in controlling endocrine cell migration and islet morphogenesis^15^. Furthermore, different PIP2 species are located in distinct domains of PM and intracellular endomembrane compartments^34^. Thus, it is possible that Syt13 apical localization and intracellular movement require its interaction with different PIP2 species.

Our findings indicate that Syt13 plays a role in vesicle trafficking and internalizing surface proteins. Moreover, the increased levels of α-tubulin and its PTM forms in Syt13-expressing cells and the functional interlink between Syt13 and MT networks suggest that MT transport is required to localize Syt13-containing vesicles to the apical and front domains. It is tempting to speculate that Syt13 is involved in the internalization of a subset of special membrane proteins (such as EGFR but not Tf receptor) rather than serving as a general endocytic protein. The fact that Syt13 does not appear to be ubiquitously expressed across all cell types also supports this notion. Lack of Syt13 impaired endocrine cell egression and correlated with the retention of α6β4 integrins levels at the front domain of egressing endocrine cells in vivo. Further, Syt13 gain-of-function reduced FA area and cell-matrix adhesion in vitro. These results suggest that Syt13 modulates cell-matrix adhesion structures by internalizing a subset of adhesion proteins such as integrin subunits. This function of Syt13 likely results in increased cell dynamics processes in the front domain of egressing endocrine cells. Therefore, in the polarized endocrine precursors residing within the epithelium, Syt13 expression in the apical domain ensures that these cells sustain an intact cell-matrix junction at the basal domain. As endocrine cells repolarize, Syt13 translocates to the front domain to lessen cell-matrix adhesion for endocrine cell egression.

The deletion of Syt13 resulted in a shift in β- to α-cell fate. It is possible that the absence of Syt13 impacts endocrine cell egression, which subsequently influences their fate decision. Along this line, lack of p120ctn in EPs has resulted in faster egression that increases differentiation into α cells^35^. Contrary to this, our findings showed that the delay in cell egression is linked with increased EP specification towards α cells. Therefore, activation of a β-cell program might require a precise timing of EP occupancy within the epithelium and that faster or slower egression induces α-cell fate. Alternatively, Syt13 might regulate endocrine fate decisions by modulating cell-matrix adhesion and the downstream signaling. Indeed, the interactions between integrins and extracellular matrix (ECM) components drive the fate of pancreatic progenitors toward ductal or endocrine lineages^36^. Finally, the lack of Syt13 might directly affect β-cell differentiation. This notion is supported by the correlation between increased Syt13 expression levels in endocrine precursors and *Fev*^+^ cells with a β-cell program. It is possible that Syt13 regulates the internalization and trafficking of upstream signaling receptors, which determine the fate of endocrine cells.

We uncovered an unexpected role for the atypical Syt13 protein as a morphogenetic driver of endocrine precursor egression. Different from the typical Syt members such as Syt1 and Syt2, Syt13 was dominantly localized to the PM of egressing endocrine cells. Syt3 has also shown similar PM localization pattern and endocytosis function in neurons^37,38^, suggesting a similar function to that of Syt13. Yet, the postnatal lethality of Syt13 KO mice indicates no redundant function for this protein by other Synaptotagmin family members. Furthermore, together with Syt1, Syt13 deletion results in the earliest lethal phenotype of all Synaptotagmin members, emphasizing the unique and critical role of this protein for organ development and function. In support of this, Syt13 is expressed in the brain^39^ and shows a protective function in motor neurons of patients with neurological disorders^40^. Thus, our findings have broader implications and may help to understand the molecular action of Syt13 in the pancreas, but also during neurogenesis and in neurological disorders. Remarkably, SYT13 is also upregulated in several cancer cell types, and its inhibition reduces cancer cell metastasis^41–43^. Our study reveals a critical function of Syt13 in epithelial cell invasion and highlights the importance of our mechanistic findings in the context of cancer cell dissemination.

## Methods

### Mouse lines

Animal studies were conducted with adherence to relevant ethical guidelines for the use of animals in research in agreement with German animal welfare legislation with the approved guidelines of the Society of Laboratory Animals (GV-SOLAS) and the Federation of Laboratory Animal Science Associations (FELASA). The study was approved by the Helmholtz Zentrum München (HMGU) Animal Welfare Body and by the Government of Upper Bavaria. Syt13 targeted genetrap *(Syt13*^*GT/GT*^*)* (EUCOMM) embryonic stem cells were aggregated with CD1 morula to generate chimeric mice. Genetrap mice were bred on a mixed background. For generation of Syt13 full KO mice, first genetrap Syt13 mice were crossed with Flpe mice to obtain floxed mice *(Syt13*^*F/F*^*)*. Then, Syt13 floxed mice were crossed with *Rosa26*^*Cre/+*^ to delete the critical exon 2 and generate the flox deleted (FD) *(Syt13*^*FD/FD*^*)* mice. Heterozygous *(Syt13*^*+/FD*^*)* intercross mice were used to obtain full knockout (KO) (Syt13 KO) embryos, which were genotyped by PCR analysis. To generate Syt13 tissue-specific conditional knockout mice, *Syt13*^*F/F*^ mice were crossed with constitutive *Tg (Neurog3-cre)C1Able/J (Ngn3*^*+/Cre*^*)*^24^ and *Ins1*^*Cre*^ *(Ins1*^*tm1(cre)Thor*^*) (Ins1*^*+/Cre*^*)* mice^25^. For endocrine cell labeling, the lineage-tracing Gt(ROSA)26^mTmG^ mouse line was used^44^. Syt13-Venus fusion mouse line, in which endogenous Syt13 is fused with the fluorescent protein Venus has been recently generated and characterized^45^. The list of used primers for mouse genotyping is provided in Supplementary Data 1. The strain of the mouse lines *Syt13*^*F/F*^*, Syt13*^*FD/FD*^, *Ngn3*^*Cre/+*^; *Syt13*^*F/FD*^, *Ins1*^*Cre/+*^; *Syt13*^*F/FD*^ was C57BL/6J and the *Syt13*^*F/F*^; Gt(ROSA)26^mTmG^, *NVF*; *Syt13*^*FD/FD*^, and Syt13-Venus fusion lines were on the mixed background (C57BL/6J × 129/SvJ). Mouse keeping was done at the central facilities at HMGU under SPF conditions in animal rooms with light cycles of 12/12 h, temperature of 20–24 °C, and humidity of 45–65%. The mice received sterile filtered water and a standard diet for rodents ad libitum.

### Cloning, cell culture and transfection

Cloning was performed using standard protocols. Different Syt13 constructs were generated using the pCAG mammalian expression vector (Supplementary Data 1). Cell transfection was performed using lipofectamine™ 2000 transfection reagent (Gibco/Invitrogen GmbH, 11668019). For generating stable cell lines, cells were transfected with linearized constructs and were treated with 1–2 µg/mL puromycin for 5 days and the surviving mixed clones were expanded.

MDCK cells (NBL-2) (Sigma, 85011435) were cultured and maintained in a standard medium (DMEM, 10% FBS, penicillin/streptomycin). For 2D staining, cells were trypsinized and then plated on µ-Slide eight-well chambers (Ibidi, 80826) followed by the experimental procedure. For 3D culture, µ-Slide eight-well chambers (Ibidi, 80821) were coated with 100% growth factor-reduced Matrigel (BD Biosciences) for 15 min at 37 °C. Single MDCK cells were resuspended in 2% Matrigel-containing medium. MDCK cysts were fixed and stained after 3–4 days.

To generate primary pancreatic epithelial cyst (PECs), E14.5 mouse embryonic pancreata were dissected and kept in 0.25% trypsin-EDTA for 15–30 min on ice and then 5 min at 37 °C^27^. After replacing the trypsin with culture medium and 30 times pipetting up and down, single-cell suspension was achieved. Single pancreatic cells were then cultured on 100% Matrigel-coated µ-Slide eight-well chambers (Ibidi, 80821) in the culture medium containing 2% Matrigel. The PECs were fixed and stained after 1–2 days.

2D pancreatic culture was established by dissecting E14.5 embryonic pancreata and incubation in 0.25% trypsin-EDTA for 15–30 min on ice followed by 5 min incubation at 37 °C. The trypsin was replaced by culture medium and partial digestion was performed by pipetting the samples up and down for ten times. Cell clusters were cultured on <10% Matrigel-coated µ-Slide eight-well chambers (Ibidi, 80821) in the condition medium.

Pancreatic explant culture was performed by dissecting E13.5 pancreata into 3–4 pieces and culturing them in µ-Slide eight-well chambers (Ibidi, 80821) coated with 50 µg/mL bovine fibronectin (Sigma, F1141). Samples were treated with 14 µM PIP3/protein binding inhibitor (PIT-1) (Abcam, ab120885) or 10 µM LY294002 (Promega, V1201). The medium was replaced with a fresh one every 24 h. Samples were fixed and stained after 2 days in culture.

### Immunostaining and imaging

Dissected embryonic pancreata or explant culture samples were fixed in 4% PFA in PBS for 2 h overnight at 4 °C. The tissues were merged in 10% and 30% sucrose-PBS solutions at RT (2 h each solution) followed by 1:1 solution 30% sucrose:tissue-freezing medium (Leica 14020108926). Afterward, they were embedded in cryoblocks using tissue-freezing medium and sections of 20 μm thickness were cut using a cryostat. Next, the samples were permeabilized (0.1% Triton, 0.1 M Glycine) for 30 min and incubated in blocking solution (10% FCS, 3% Donkey serum, 0.1% BSA and 0.1% Tween-20 in PBS) for 1 h at room temperature (RT). Then, the primary antibodies (Supplementary Data 1) diluted in the blocking solution were added to the samples overnight at 4 °C. After washing with PBS, they were stained with secondary antibodies (Supplementary Data 1) diluted in the blocking solution for 3–5 h at RT. The samples were then incubated with 4′, 6-diamidin-2-phenylindol (DAPI) for 30 min, followed by washing with PBS and embedding in commercial medium (Life Tech., ProLong Gold).

2D and 3D cultures of cell lines or primary pancreatic cells were fixed in 4% PFA (12 min at 37 °C) followed by 10 min permeabilization (100 mM Glycine and 0.2% Triton X-100) at RT. After 3x washing, cells were incubated with blocking solution for 30 min at RT and then incubated with primary antibodies (Supplementary Data 1) for 1–3 h at RT. After washing, cells were incubated with secondary antibodies (Supplementary Data 1) for 1 h at RT and DAPI was added and samples were embedded.

Images were obtained with a Leica microscope of the type DMI 6000 using LAS AF software or Zeiss LSM880 inverted confocal microscope. Images were analyzed using LAS AF and ImageJ software programs.

### Whole pancreata clearing and mount immunostaining

The dissected pancreata was fixed in 4% PFA for 24 h at 4 °C. After three times rinsing with PBS, single pancreas were bleached in Dent’s bleach (1:4, DMSO:MeOH) at 4 °C for 24 h. Bleached pancreas was then rinsed five times using MeOH followed by fixation using Dent’s Fix solution (1:2, H_2_O_2_:Dent’s Fix) for 24 h at 4 °C. Before proceeding to the immunostaining, the bleached pancreata were washed three times (1 h) with PBS followed by blocking (donkey blocking solution) for 3 h at RT. Primary antibodies were then applied and incubated for 5 days on a standing shaker at RT. After three washes (1 h) with PBS, the secondary antibodies were incubated for 1.5 days on a standing shaker at RT. Nucleus staining with DAPI was performed after three times rinsing with PBS and incubated for 1 h at RT. In order to fully clear the pancreas, we incubated the pancreas in a 1:1 solution of PBS and MeOH for 10 min, followed by three times (20 min) washing with MeOH. Then, we replaced half volume of the MeOH with BABB solution (1:2, Benzyl alcohol:benzyl benzoate) and after 10 min of incubation, the pancreas were immerse in 100% BABB solution. Finally, the samples were mounted in coverslips using Secure-SealTM spacers and BABB solution. For whole-mount staining, images were acquired using the 63× objective and “tile scan functiopn” of LAS AF software. Final movies were generated using Imaris imaging software (Bitplane).

### Embryonic proto-islet cluster isolation

In order to isolate proto-islets from E18.5 embryos, we adapted the protocol reported by Huang and Gu for single pancreata^46^. The pancreata were dissected and cut in small pieces, and were individually incubated in 500 µL collagenase type IV (3 mg/mL) for 5–10 min at 37 °C. We performed mechanical disruption every 2 min during the incubation. The full disruption of the tissue was monitored, and the digestion was stopped using 500 µL of G-Solution (HBSS, 1% Pen/Strep, 1% BSA). Samples were then centrifuged (500 × *g* for 10 s) and the supernatant was carefully discarded for three times. The pellet was then resuspended in G-Solution and islets were handpicked using a dissecting microscope. The islets were incubated overnight in culture medium (RPMI 1640, 1% Pen/Strep, 10% inactivated fetal bovine serum) and then were used to extract protein.

### Cell sorting and quantitative PCR (qPCR) analysis

Embryonic pancreata from NVF; Control and NVF; Syt13 KO at E15.5 were dissected. Next, individual pancreata were kept in 0.25% Trypsin for 5 min on ice and then incubated at 37 °C for 10 min. The single-cell samples were then centrifuged at 100 × *g* for 5 min at 4 °C. Five microliters anti-mouse CD326 (EpCAM) PE (eBioscience, 12-5791-81) and rat IgG2a K isotype control (eBioscience, 12-4321-42) were used for 1 × 10^6^ cells in 100 µL total volume. After staining for 15 min at 4 °C, the cells were washed with PBS and stained with DAPI for 5 min to detect dead cells. The samples were then washed and resuspended in the FACS buffer (PBS, 1% BSA, 0.5 mM EDTA) and loaded for FACS sorting. The gating strategy was as follows: main population> single cells > living cells (DAPI negative)> EpCam^+^ (PE^+^) and Ngn3^+^ (FITC^+^) cells. The cells were collected in Qiazol (Qiagen, 79306). After RNA isolation, a MessageAmp™ II aRNA Amplification Kit (Thermo Fisher Scientific) was utilized to maximize the yield per sample. The procedure for the amplification was undertaken as stated in the kit’s protocol. Briefly, as first step a cDNA amplification synthesis by reverse transcription was prepared, followed by cDNA purification. Next, an in vitro transcription reaction was prepared to generate multiple copies of amplified RNA (aRNA) from the double-stranded cDNA templates providing aRNA. Finally, the aRNA was purified and quantified. qPCR analysis was done using TaqMan™ probes (Life Technologies, Supplementary Data 1) and 25 ng cDNA per reaction. Each reaction consisted of 4.5 µL cDNA in nuclease-free water, 5 µL TaqMan™ Advanced master mix (Life Technologies) and 0.5 µL TaqMan probe™. The qPCR was performed using Viia7 (Thermo Fisher Scientific). Ct-values were normalized among samples, transformed to linear expression values, normalized on reference genes and on control samples.

Relative expression (gene) = (2Ct (mean genes) − Ct (gene))/(2Ct (mean references) − Ct (reference))

Normalized expression (gene) = Relative expression (gene)/Relative expression control (gene).

### Western blotting

Protein samples were loaded on SDS-PAGE gel and then were transferred to Nitrocellulose membrane (Bio-Rad, Cat. Nr. 1620112). After blocking for 1 h at RT, membranes were incubated with the primary antibodies (Supplementary Data 1) at 4 °C overnight followed by 3× washing and then incubating with the secondary antibodies (Supplementary Data 1) for 1 h at RT. Next, membranes were incubated with Pierce ECL western blotting substrate (Thermo Fisher Scientific) or SuperSignal West Pico PLUS Chemilumineszenz-Substrat (Thermo Fisher Scientific) and the signals were detected by enhanced chemiluminescence. The uncropped and unprocessed scans of the blots are provided (Fig. S10).

### Surface biotinylation

1 × 10^5^ MDCK cells stably expressing Syt13-TAP recombinant proteins were cultured in six-well plates in grocontrolh medium for 3 days at 37 °C. The cells were washed 3× with ice-cold PBS pH 8.0. Cells were then incubated with 500 µL of 2 mM EZ-link Sulfo-NHS-Biotin (Thermo Scientific, 21425) in PBS for 15 min on ice. Samples were then washe 3x with PBS containing 100 mM glycine and were lysed with lysis buffer (1× TBS, 0.5% NP40, protease inhibitor) for 15 min on ice. After centrifugation, 30 µL of supernatants were stored as input and the rest were incubated with the lysis buffer-prewashed G-sepharose beads (50 µL of 50% slurry per each reaction; Protein G-Sepharose 4 Fast Flow, GE Healthcare) for 3 h at 4 °C while rotating. After centrifugation, the supernatants were incubated with lysis-buffer prewashed high capacity NeutrAvidin agarose resin beads (50 µL of 50% slurry per each reaction) (Thermo Scientific, 29202) overnight at 4 °C rotating. The beads were then washed 3x with the washing buffer (1× TBS, 0.1% NP40) and 40 µL sample buffer were added. Together with the input fractions, the samples were applied to western blotting.

### Immunoprecipitation using BioID approach

1 × 10^6^ MDCK cells stably expressing Syt13 or Syt13-BirA recombinant proteins were cultured in 14 cm^2^ dishes in grocontrolh medium for 3 days at 37 °C. The medium was replaced with a fresh growth medium containing 50 µM biotin and after 16 h each dish was lysed with 1 mL lysis buffer (50 mM Tris buffer pH 7.5, 0.5% Sodiumdesoxycolate, 150 mM NaCl, 1% NP40, 0.1% SDS) and kept on ice. In parallel, 240 µL Pierce™ High Capacity NeutrAvidin™ Agarose beads (ThermoFisher Scientific, Cat: 29202) were washed 3x with the lysis buffer and resuspended in 1 mL lysis buffer. Next, the cell lysates were centrifuged for 30 min at 6790 × *g* at 4 °C and 3 mL supernatant for each condition was collected. Ninety microliters of the samples were stored as the input samples and the rest were mixed with 300 µL beads-lysis buffer in the 15 mL falcon tubes rotating for 3 h at RT. Next, samples were centrifuged for 5 min at 9 × *g* at 4 °C. Supernatants were discarded and the beads were used for western blotting or mass spectrometry as follows.

For western blotting, the beads were washed 3× with the washing buffer (50 mM Tris buffer pH 7.5, 0.5% Sodiumdesoxycolate, 150 mM NaCl, 1% NP40, 0.5% SDS) and 2x with 1x TBS. The beads were then resuspended in 90 µL sample buffer, heated for 5 min at 95 °C. After cooling down on ice the samples were centrifuged and the supernatants were collected as the IP sample, which together with the input samples were applied to western blotting.

For mass spectrometry, 500 µL TBS was added to the beads and mixed rigorously. After centrifugation at 7000 × *g* for 30 second supernatants were removed and the washing was repeated two more times. Sixty microliters elution buffer (2 M urea, 50 mM Tris-HCl pH 7.5 and 5 μg/mL Trypsin (SIGMA; T6567-5X20UG)) (twice as the bead volume) was added and samples were incubated for 1 h at 27 °C under constant agitation (800 rpm in a thermo mixer) followed by centrifugation. Supernatants were collected in a fresh Eppendorf tube. The beads were then resuspended with 60 µL of the elution buffer (containing 2 M urea, 50 mM Tris-HCl pH 7.5 and 1 mM DTT), centrifuged and the supernatant was collected and pooled with the previous one. This last step was repeated and 180 µL total volume per each reaction was obtained. Samples were left at RT to continue to digest overnight and were stored at −80 °C for further mass spectrometry procedure.

### Mass spectrometry

Affinity purified eluates were precipitated with chloroform and methanol followed by trypsin digestion^47^. LC–MS/MS analysis was performed on Ultimate3000 nanoRSLC systems (Thermo Scientific) coupled to an Orbitrap Fusion Tribrid mass spectrometer (Thermo Scientific) by a nanospray ion source. Tryptic peptide mixtures were injected automatically and loaded at a flow rate of 30 μL/min in 0.1% trifluoroacetic acid in HPLC-grade water onto a nano trap column (300 μm i.d. × 5 mm Pre column, packed with Acclaim PepMap100 C18, 5 μm, 100 Å; Thermo Scientific). After 3 min, peptides were eluted and separated on the analytical column (75 μm i.d. × 25 cm, Acclaim PepMap RSLC C18, 2 μm, 100 Å; Thermo Scientific) by a linear gradient from 2% to 30% of buffer B (80% acetonitrile and 0.08% formic acid in HPLC-grade water) in buffer A (2% acetonitrile and 0.1% formic acid in HPLC-grade water) at a flow rate of 300 nl/min over 117 min. Remaining peptides were eluted by a short gradient from 30% to 95% buffer B in 5 min. Analysis of the eluted peptides was done on an LTQ Fusion mass spectrometer. From the high-resolution MS pre-scan with a mass range of 335 to 1500, the most intense peptide ions were selected for fragment analysis in the orbitrap depending by using a high speed method if they were at least doubly charged. The normalized collision energy for HCD was set to a value of 27 and the resulting fragments were detected with a resolution of 120,000. The lock mass option was activated; the background signal with a mass of 445.12003 was used as lock mass^48^. Every ion selected for fragmentation was excluded for 20 seconds by dynamic exclusion. MS/MS data were analyzed using the MaxQuant software (version 1.6.1.0)^49,50^. As a digesting enzyme, Trypsin/P was selected with maximal 2 missed cleavages. Cysteine carbamidomethylation was set for fixed modifications, and oxidation of methionine and N-terminal acetylation were specified as variable modifications. The data were analyzed by label-free quantification with the minimum ratio count of 3. The first search peptide tolerance was set to 20, the main search peptide tolerance to 4.5 ppm and the re-quantify option was selected. For peptide and protein identification the human subset of the SwissProt database (release 2014_04) was used and contaminants were detected using the MaxQuant contaminant search. A minimum peptide number of 2 and a minimum length of 7 amino acids was tolerated. Unique and razor peptides were used for quantification. The match between run option was enabled with a match time window of 0.7 min and an alignment time window of 20 min. The statistical analysis including ratio, t-test and significance A calculation was done using the Perseus software (version 1.6.2.3)^51^.

Interaction candidates from mass spectrometry were used to gain insights into the biological processes involved with Syt13. We used Metascape^52^ to perform the pathway enrichment analysis.

### Transferrin and EGF uptake assay

Internalization of transferrin (Life Technologies, T13342) and EGF (Life Technologies, E13345) coupled to Alexa Fluor 488 was performed according to the manufacturer’s instructions. Briefly, MDCK cells were seeded in μ-slide 8 well chambers (Ibidi, 80826) one day prior to the experiment. Cells were washed three times with ice-cold PBS and incubated in uptake medium (DMEM, 2% BSA, 20 mM HEPES, pH 7.5) for 10 min on ice. Next, cells were incubated with uptake medium containing 25 μg/mL Alexa Fluor 488–Transferrin or 5 μg/mL Alexa Fluor 488–EGF for the indicated time points at 37 °C, followed by fixation with 4% PFA, immunostaining and confocal microscopy.

Cells were automatically segmented using Cellpose^53^ using DAPI and E-Cadherin signal and mean fluorescence intensity per cell was measured using ImageJ.

### Time-lapse imaging of pancreatic cells in 2D culture

One day after culturing of primary pancreatic cells, images were acquired in time intervals of 15 min. Z-stacks of different sizes were acquired in order to capture the whole cell (parameters vary slightly in some experiments). Cells were automatically segmented using Cellpose^53^ by plasma membrane mTmG fluorescence. Based on this mask manual tracking of cells was performed using the TrackMate plugin for ImageJ, allowing for automatic computation of morphological features (cell area, circularity and solidity) and track analysis (cell speed). In some cases, cells in subsequent frames were not able to be segmented properly. Here, cell tracks were manually merged for frames identifying individual cells.

### Time-lapse imaging of Syt13-containing vesicle movement

MDCK cells overexpressing Syt13-Venus were cultured on μ-slide eight-well chambers (Ibidi, 80821). Time-lapse imaging was performed on a Leica SP5 inverted confocal microscope. Images of cells were acquired in time intervals of 3 s. Syt13-positives vesicles were manually tracked using the MtrackJ plugin for ImageJ^54^. At least five cells (10 tracks per cell) per condition for each replicate were quantified.

### Time-lapse imaging of lysosomal movement

MDCK cells overexpressing Syt13-Venus were cultured on μ-slide 8 well chambers (Ibidi, 80821). Cells were starved for 30 min in serum-free medium and were incubated with LysoTracker Red DND-99 (Invitrogen, L7528; 10:1000) to label lysosomes. After chasing with prewarmed growth medium for 60 min, time-lapse imaging was performed on a Zeiss LSM880 inverted confocal microscope with AiryScan2 module. Images of cells were acquired in time intervals of 350 msec using AiryScan2 Fast Mode.

### Measurement of focal adhesion area

MDCK cells were seeded in μ-slide eight-well chambers (Ibidi, 80826) one day prior to the experiment. Cells were starved in serum-free medium for 2 h or incubated with 10 μM nocodazole in serum-free medium for 2 h at 37 °C and subsequently fixed with 4% PFA and processed for confocal microscopy. Cell were immunostained with paxillin as the marker for focal adhesion and images were acquired using Leica SP5 microscope. All steps of image processing were carried out using ImageJ. To generate a mask for quantification raw images were processed as follows: Background was subtracted using the sliding paraboloid function (size: 50 px). Top-hat filtering (size 2 px), gaussian blur (size 1 px) and subsequent thresholding was applied to generate a binary image of focal adhesion. The “Analyze Particle” function (size: 0.3—infinity; circularity: 0.00–0.99) was used to identify focal adhesion. This mask was overlaid on raw image files to measure the fluorescence signal. For final quantification of focal adhesion, the integrated density of paxillin fluorescence was normalized to the total cell area per image. At least 10 confocal images per condition for each replicate were quantified.

### Cell detachment assay

MDCK cells were seeded into 6-well plates until they reached full confluency. Cells were treated with 1 mL 0.25% trypsin-EDTA for 4 min at 37 °C, followed by adding 1 mL culture media containing 10% BSA. The mixed media and trypsin were pipeted up and down 5 time to the cell layer to obtain the detached cells, considered as the fraction A (Fig. S9k). The remaining attached cells were washed with PBS and incubated with 1 mL 0.25% trypsin-EDTA for 10 min at 37 °C. All remaining cells were recovered from the plate and were considered as the fraction B. To define the cell detachment rate, the cell number in fraction A was divided to the cell number in fraction B (Fig. S9k).

### Purification of recombinant domains of Syt13 protein variants

Three different constructs expressing recombinant Syt13 protein variants fused with Myc-tag were generated using the pGEX-6P-1 vector (Supplementary Data 1). These included Syt13-C2AB (aa 39–426 of mouse Syt13), Syt13-C2A (aa 39 to 282 of mouse Syt13), Syt13-C2B (aa 289–426 of mouse Syt13). Recombinant protein production was carried out in Escherichia coli BL21 (DE3). Bacterial cells were grown in a total of 700 mL LB medium supplemented with ampicillin (100 µg/mL) to reach an OD A600 of 0.7 at 37 °C, induced with 0.5 mM isopropyl β-D-1-thiogalactopyranoside, and incubated for 3-6 h at 25 °C. Bacterial lysate was prepared by sonication in lysis buffer (50 mM Tris pH 8.0, 300 mM NaCl, 4 mM DTT, 2 mM EDTA), followed by three passes through a Emulsiflex C3 homogenizer (Avestin Inc., Ottawa, Canada). After removal of cell debris by centrifugation for 15 min at 20,000 × *g* at 4 °C, the supernatant was incubated with 5 mL Glutathione Sepharose 4B (GE Healthcare) for 1 h at 4 °C in batch mode under gentle rotation. Beads were packed into the column, washed with 20 column volumes (CV) wash buffer 1 (50 mM Tris pH 8.0, 750 mM NaCl, 4 mM DTT, 2 mM EDTA, 50 mM KCL, 10 mM MgCl2, 1 mM ATP) and subsequently with 20 CV wash buffer 2 (50 mM Tris pH 8.0, 750 mM NaCl, 2 mM DTT). Beads were then incubated with recombinant GST-tagged human rhino- virus 3 C protease (50 µg per CV) for 16-18 h at 4 °C and the protein was eluted with 6 CV elution buffer (50 mM Tris pH 8.0, 150 mM NaCl, 1 mM DTT). The eluted protein was concentrated using ultrafiltration units with 10 kDa regenerated cellulose membrane (Amicon Ultra, Merck Millipore) and subsequently loaded on an equilibrated (50 mM Tris pH 8.0, 150 mM NaCl, 1 mM DTT, 5% glycerol) Superdex 75 10/300 GL column (GE Healthcare) for size exclusion chromatography. Protein purity was assessed by SDS-PAGE (NuPAGE gels; 12% bis-tris gels, and MOPS buffer both Invitrogen). Dynamic Light Scattering (Zetasizer nano ZS, Malvern Instruments, Worcestershire, UK) was used to confirm monomeric state. For protein quantification Bradford assay1 or BCA protein assay kit (Pierce, Thermo Fisher Scientific) was used.

### Preparation of large unilamellar vesicles (LUVs)

*1*-*palmitoyl*-*2*-*oleoyl phosphatidylcholine* (POPC) and phosphoinositides were purchased from Avanti Polar Lipids, Alabaster; cholesterol was purchased from Sigma-Aldrich, Germany. POPC and chloroform stocks were dissolved in chloroform, phosphoinositides in chloroform:methanol:water 1:2:0.8. The fatty acid distribution consists of C18:1, mimicking unsaturated species found within organisms. Lipid concentration and stability of the stocks were validated by a total phosphorus assay^55^ and thin-layer chromatography (TLC) at regular basis. For vesicle preparation, lipids were mixed in desired mol % ratio (POPC/cholesterol/phosphoinositide 65/30/5 mol %, POPC/cholesterol 70/30 mol %) and dried under nitrogen gas stream, followed by incubation under vacuum for 2–16 h to remove organic solvents. The dried lipid film was re-hydrated in liposome buffer (10 mM Hepes, 150 mM NaCl, pH 7.4) to a final concentration of 1 mg/mL, for 15 min at 300 rpm. Multilamellar vesicles were then subjected to 10 cycles of freezing in liquid nitrogen and subsequent thawing in a heating block at 18 °C. The vesicle solution was extruded 21 times through a 100 nm diameter polycarbonate membrane (Whatman® Nuclepore, Fisher Scientific) using an extrusion kit (Avanti Polar Lipids, Alabaster). For quality control, vesicles were subjected to dynamic light scattering (DLS) and zeta potential measurements (see following section). Lipid composition and stability of vesicles were analyzed by TLC.

### Size and zeta potential measurements

Size and zeta potential of Large Unilamellar Vesicles (LUV), and protein size were determined using a Zetasizer Nano ZS (Malvern Instruments, UK). For size measurements LUVs were diluted to a final concentration of lipids ranging from 0.08 to 0.4 mg/mL. Size of proteins was measured at a concentration above 0.5 mg/mL in buffer (50 mM Tris, 150 mM NaCl, 1 mM DTT, pH 8.0). Importantly, all buffers were filtered (PVDF, 0.45 µm, Millipore) prior to use. Zeta-potential of LUVs was measured at a total lipid concentration of 0.08 mg/mL in filtered (PVDF, 0.45 µm, Millipore) water.

### Thin layer chromatography

TLC was used to validate lipid composition of lipid stocks and LUVs. LUVs were extracted with choloroform:methanol:aceton:1 N HCl (2:1:0,5:0,1 v/v), where the organic phase was dried under nitrogen stream and dissolved in 20 μL of extraction solution. Silica-coated TLC plates (HPTLC Silica Gel 60, 10 × 10 cm, Merck Darmstadt) were pre-treated with 1% potassium oxalate in methanol:water (2:3 v/v) for 12-16 h and dried under vacuum at RT. The extracts were spotted on TLC plates and placed in a closed glass chamber with chloroform:acetone:methanol:acetic acid:water (46:17:15:14:8 v/v). Subsequently, the TLC plate was air-dried and lipids were visualized by spraying the plate with primuline (Sigma-Aldrich) solution (5% primuline in acetone:water 8:2 v/v), which was then scanned on a Typhoon 9410 imager using 457 nm laser (GE Healthcare).

### Electrochemiluminescence-based immunoassay

The binding assay was performed using Meso Scale Discovery 384 well high bind plates^56^. All binding experiments were carried out at 22 °C. Liposomes (2 μL) were passively adsorbed on the electrode surface for 1 h, and residual sites on the surface were blocked for 1 h with 0.25% porcine gelatin (Sigma-Aldrich) in TRIS buffer (50 mM Tris, 150 mM NaCl, pH 8.0). After three washing steps with TRIS buffer, serial dilutions of recombinant protein in blocking buffer was added to the respective wells and incubated for 2 h. Unbound protein was removed, and anti-c-myc antibody (clone 9E10, Santa Cruz Biotechnology) at 1.25 µg/mL concentration in 0.25% porcine gelatin was applied for 1 h followed by three subsequent wash steps with TRIS buffer. For detection, a secondary anti-mouse antibodies labeled with Sulfo-TAG (Meso Scale Discovery) was used at 1.25 μg/mL in blocking buffer for 1 h in the dark. Free secondary antibody was washed off, and reading buffer (surfactant-free reading buffer from Meso Scale Discovery) was added. The readout was performed on a Meso Scale Discovery SECTOR Imager 6000 chemiluminescence reader. Data were analyzed with GraphPad Prism 6.07. First, signal from PC/cholesterol (70/30 mol %) vesicles was subtracted as a background. Next, a non-linear curve fitting was applied, and the binding kinetics were calculated using one site—specific binding fitting.

### Image quantification

To quantify the rate of egression of endocrine cells, confocal images were taken from various areas of the pancreas. We counted the ratio (in percentage) of Nkx6-1^high^/Sox9^-^ cells within or directly in contact with the epithelium to the total Sox9^+^ cells. LAS-AF software was used for analysis.

The direct attachment of proto-islets to the epithelium was measured using confocal imaging of different pancreatic regions. We quantified the direct attachment area between the epithelium and proto-islets relative to the total area of the proto-islet periphery. Areas were detected via “wand tracing tool”, supervised by adjusting the tolerance level based on the quality of the staining. Surfaces of contact were manually drawn. The analysis was conducted using Fiji ImageJ. Average of 4 Z-stacks (2 μM) per picture were used for each data point.

The number of Ngn3^+^ cells was quantified from confocal images taken from randomly selected pancreatic regions using automatic nuclei counts with IMARIS software (Bitplane).

Using Leica LAS-AF software, we counted hormone-expressing cells in pancreatic sections to quantify the α- to β-cell ratio.

The apical localization of Syt13 was determined by dividing the signal intensity of the recombinant Syt13 protein variants at the apical domain by the total cytosolic signal. The analysis was conducted using LAS-AF software.

The rate of endocrine cell egression in the explant culture was quantified using confocal images from different pancreatic areas. We measured the percentage of GFP^+^ cells within the epithelium compared to the total epithelial cells. LAS-AF was used to analyze the data.

Detection, colocalization, and quantification of Syt13, microtubules, and LysoTracker-positive vesicles were performed using ComDet v.0.5.3 plugin for ImageJ (https://github.com/ekatrukha/ComDet). Cells were manually segmented based on E-Cadherin staining and according channels were used for colocalization analysis. Due to high expression of Syt13 in trans-Golgi compartments, the nuclear and perinuclear area were manually cropped and excluded in the analysis.

To quantify the levels of α6 and β4 integrin subunits at the distal domain of endocrine cells, confocal images from different pancreatic areas were used. Signal intensity at the distal domain of endocrine cells was divided by signal intensity at the basal domain of nearby epithelial cells. LAS-AF was used to perform the analysis.

### ScRNA-seq data sources and analysis

For all scRNA-seq data analyses python 3.7.6 and the Scanpy package (v1.4.4) (https://github.com/theislab/scanpy) were used^57^. Processed and normalized scRNA-seq data and cell annotations of mouse embryonic pancreatic cells^22^ were downloaded from GEO (accession number: GSE132188). The mouse data include cells from four embryonic stages (E12.5–E15.5) from the pancreatic epithelium enriched for endocrine lineages. To classify cells as *Syt13*^high^ and *Syt13*^low/−^, we applied a threshold of 1 to *Syt13* expression values (normalized counts). To infer lineage relationships between *Syt13*^high^ and *Syt13*^low/−^ precursors and *Fev*^+^ and hormone^+^ clusters partition-based graph abstraction (PAGA)^58^ was performed as implemented in Scanpy *tl.paga* with a threshold of 0.05 for cluster connectivity. As input to PAGA, we recomputed the single-cell nearest neighbor-graph (kNN) on the 50 first principal components computed on top highly variable genes (*pp.highly_variable* with default parameters) and a local neighborhood size of 15. For differential expression testing a t-test as implemented in tl.rank_genes_groups was used. Genes expressed in <20 cells of the subset of cells used for testing were excluded. For GO term enrichment the top ranked genes sorted based on the t-test statistic were used (top 500 for endocrine precursors clusters, top 300 for FEV+ clusters). GO term enrichment was performed with the gseapy (v0.10.2) implementation of EnrichR^59^.

Processed and normalized scRNA-seq data and cell annotations of human in vitro stem cell differentiation^23^ were downloaded from GEO (accession number GSE114412). The human data contains cells sampled from stage 5 of the differentiation protocol.

Raw scRNA-seq data from human fetal pancreas were downloaded from the data visualization center descartes (https://descartes.brotmanbaty.org/bbi/human-gene-expression-during-development/)^26^, and loaded into R to convert the rds-file to an AnnData object for downstream analysis with the rpy2 (v3.3.5, https://github.com/rpy2/rpy2) and anndata2ri (v1.0.4, https://github.com/theislab/anndata2ri) python packages. Raw counts were normalized using total count normalization and log-transformed (log(count+1)). For the per cell normalization factor highly expressed genes in a cell were not considered (i.d. the parameter *exclude_highly_expressed* was set to *True* in *pp.normalize_total*). To identify endocrine lineage populations we iteratively clustered and annotated cells with the louvain clustering method as implemented in the louvain-igraph package (v0.7.0, https://github.com/vtraag/louvain-igraph)^60^ and adopted by Scanpy in *tl.louvain* (for details see available analysis notebook). For each round of clustering a single-cell kNN using 15 neighbors was computed on the 50 first principal components of the expression matrix of the 4000 top highly variable genes. Genes expressed in <10 cells were excluded before normalization and clustering. Clusters were annotated based on marker genes and merged if expressing the same markers. Mesenchymal clusters were distinguished from epithelial cells (endocrine lineage) based on the expression of the markers *EPCAM* and *VIM*, and excluded. *FEV*+ endocrine precursors were annotated using *FEV* as a marker. *NEUROG3* was not detected. Endocrine lineage precursors were annotated based on co-expression of *FEV* and the transcription factors *ARX* for α-cell precursors and *PAX4* for β-cell precursors. Hormone+ clusters were annotated with the hormones *GCG* for α cells, *INS* for the β cells, *SST* for δ cells, and *GHRL* for Ɛ cells.

### Statistics and reproducibility

All statistical analysis was performed on GraphPad prism 9 version 9.0.1. The *p* value is presented in each graph. The *n* number for each graph is provided in the corresponding figure legend.

### Reporting summary

Further information on research design is available in the Nature Research Reporting Summary linked to this article.

## Supplementary information

Supplementary Information

Description of Additional Supplementary Files

Supplementary Data 1

Supplementary Data 2

Supplementary Data 3

Supplementary Movie 1

Supplementary Movie 2

Supplementary Movie 3

Supplementary Movie 4

Supplementary Movie 5

Supplementary Movie 6

Reporting Summary

## Data Availability

The mass spectrometry proteomics data generated in this study have been deposited to the ProteomeXchange Consortium via the PRIDE^61^ partner repository with the dataset identifier PXD026699. The data are available under the following link: http://www.ebi.ac.uk/pride/archive/projects/PXD026699. Source data are provided with this paper.

## Source data

Source Data

## Code availability

Custom code for all analyses of scRNA-seq data are available as jupyter notebooks in a github repository: https://github.com/theislab/2022_Bakhti_pancreas_Syt13^62^. Processing and analyses of publicly available count matrices were carried out using custom scripts run as jupyter notebooks (jupyterlab v2.1.3), the scRNA-seq analysis suite scanpy v1.4.4 and the following python packages: anndata v0.7.1, umap v0.3.10, numpy v1.17.0, scipy v1.3.1, pandas v0.25.1, scikit-learn v0.21.3, statsmodels v0.10.1, python-igraph v0.7.1, louvain v0.6.1, seaborn v0.11.1, matplotlib v3.0.3, xlsxwriter v1.3.7, rpy2 v3.3.5, anndata2ri v1.0.4, gseapy v0.10.2.
