# Supplementary material for: Synaptotagmin-13 orchestrates pancreatic endocrine cell egression and islet morphogenesis
Source: Nat Commun. 2022 Aug 4;13:4540. doi: 10.1038/s41467-022-31862-8 (PMC9352765; doi:10.1038/s41467-022-31862-8)
Supplement: Supplementary file 1 — Supplementary Information [file 41467_2022_31862_MOESM1_ESM.pdf]

Synaptotagmin-13 orchestrates pancreatic endocrine cell egression and islet morphogenesis

Mostafa Bakhti, Aimée Bastidas-Ponce, Sophie Tritschler, Oliver Czarnecki, Marta Tarquis-Medina, Eva Nedvedova, Jessica Jaki, Stefanie J. Willmann, Katharina Scheibner, Perla Cota, Ciro Salinno, Karsten Boldt, Nicola Horn, Marius Ueffing, Ingo Burtscher, Fabian J. Theis, Ünal Coskun, Heiko Lickert

These authors contributed equally (M.B and A.B.P)

Correspondence: mostafa.bakhti@helmholtz-muenchen.de (M.B.), heiko.lickert@helmholtz-muenchen.de (H.L.)

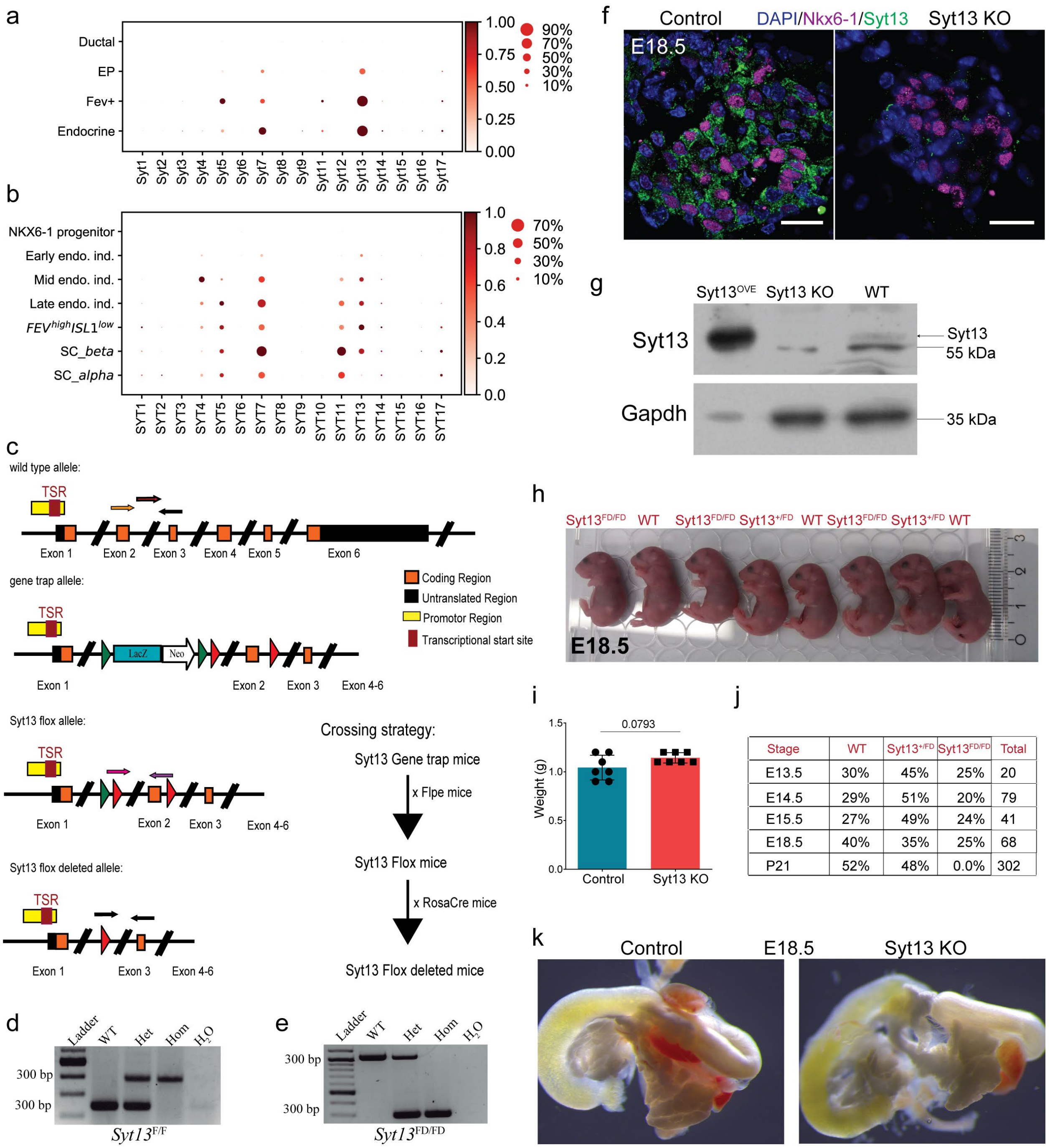

Supplementary Fig. 1. Generation and characterization of Syt13 KO mice. (a, b) Dot plots showing the expression of Syt family members during mouse (a) and human in vitro (b) endocrinogenesis in scRNA-seq data. (c) Schematic representation of Syt13 allele and the strategy for generation Syt13 Flox-deleted (FD) allele to generate *Syt13*$^{FD/FD}$ (Syt13 knockout (Syt13 KO)) mice. (d, e) PCR analyses for genotyping *Syt13*$^{F/F}$ and full-body *Syt13*$^{FD/FD}$ mice. (f) IHC of pancreatic sections confirms deletion of Syt13 protein in

Syt13 KO mice. Scale bar 20 μm. (g) Western blot analysis of isolated proto-islet clusters from WT and Syt13 KO pancreata at E18.5. Lysate from $Syt13^{OVE}$ MDCK cells was used as the positive control. The specific band corresponding to Syt13 is shown by arrow. The lower band is an unspecific signal for the used antibody. (h) Gross morphological analysis and (i) the weight of Syt13 KO and control embryos at E18.5. n=7 embryos from 4 litters. Two-sided t-test. (j) Mendelian ratio of offspring resulting from the cross between heterozygous *($Syt13^{+/FD}$)* mice shows no embryonic but postnatal lethality. (k) Gross morphological analysis of Syt13 KO and control pancreata at E18.5. Representative pictures from 3 independent pancreata (d, e, f, g). Two-sided t-test. Data are represented as mean ± SD. Source data are provided as a Source Data file.

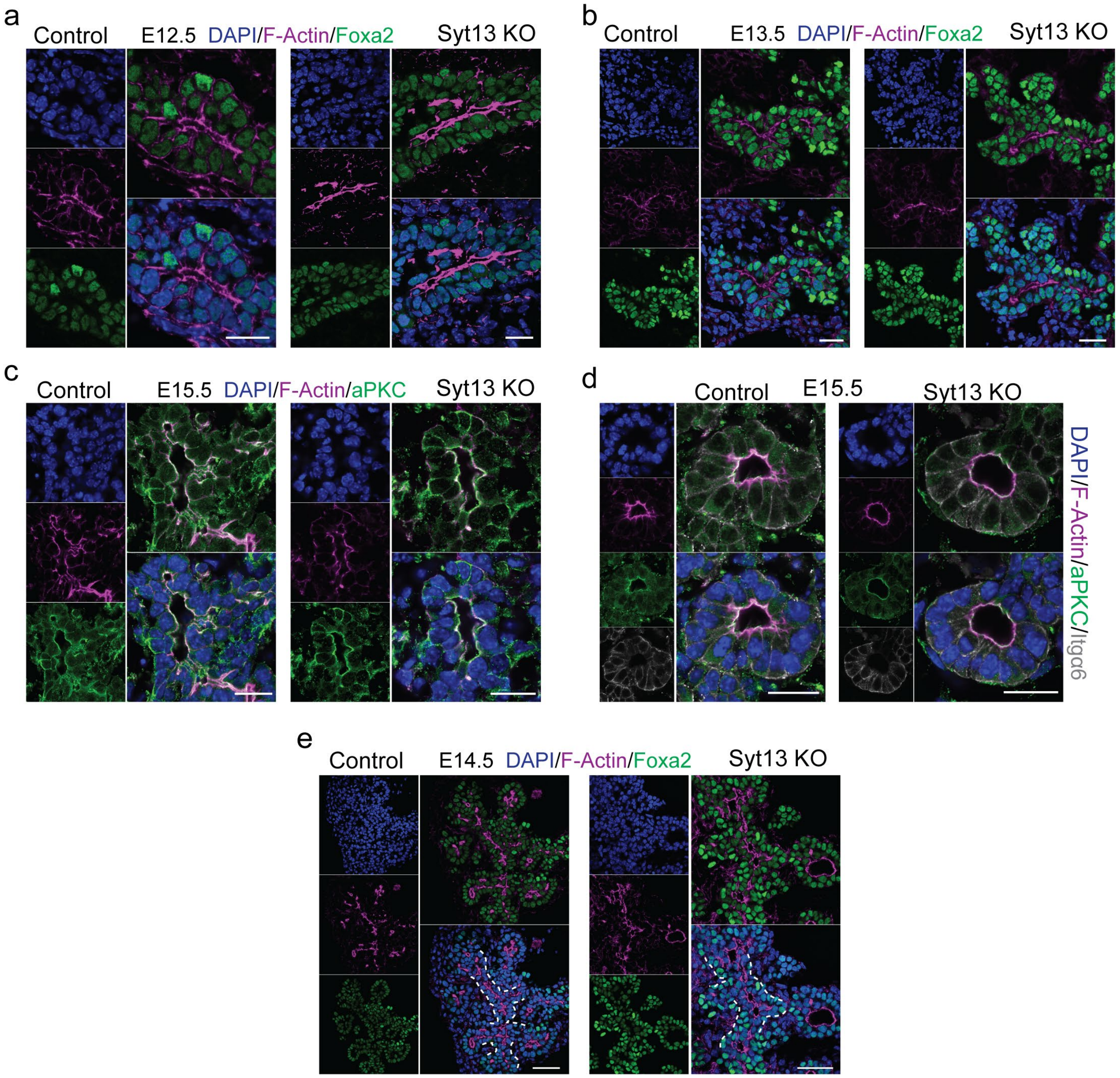

Supplementary Fig. 2. Epithelium architecture and polarity in Syt13 KO mice. (a, b) Comparable epithelial organization between control and Syt13 KO pancreata at E12.5 and E13.5. (c) Normal apical-basal polarity of ductal epithelium in control and Syt13 KO pancreata. (d) Normal apical-basal polarity of acinar cells in Syt13 KO pancreata. (e) A multi-layer epithelium appears in Syt13 KO pancreata. The extra layer is composed of Foxa2^{high} cells. White dashed lines indicate epithelium. Representative pictures from 4 independent pancreata (a-e). Scale bar 20 μm (a-d); 40 μm (e).

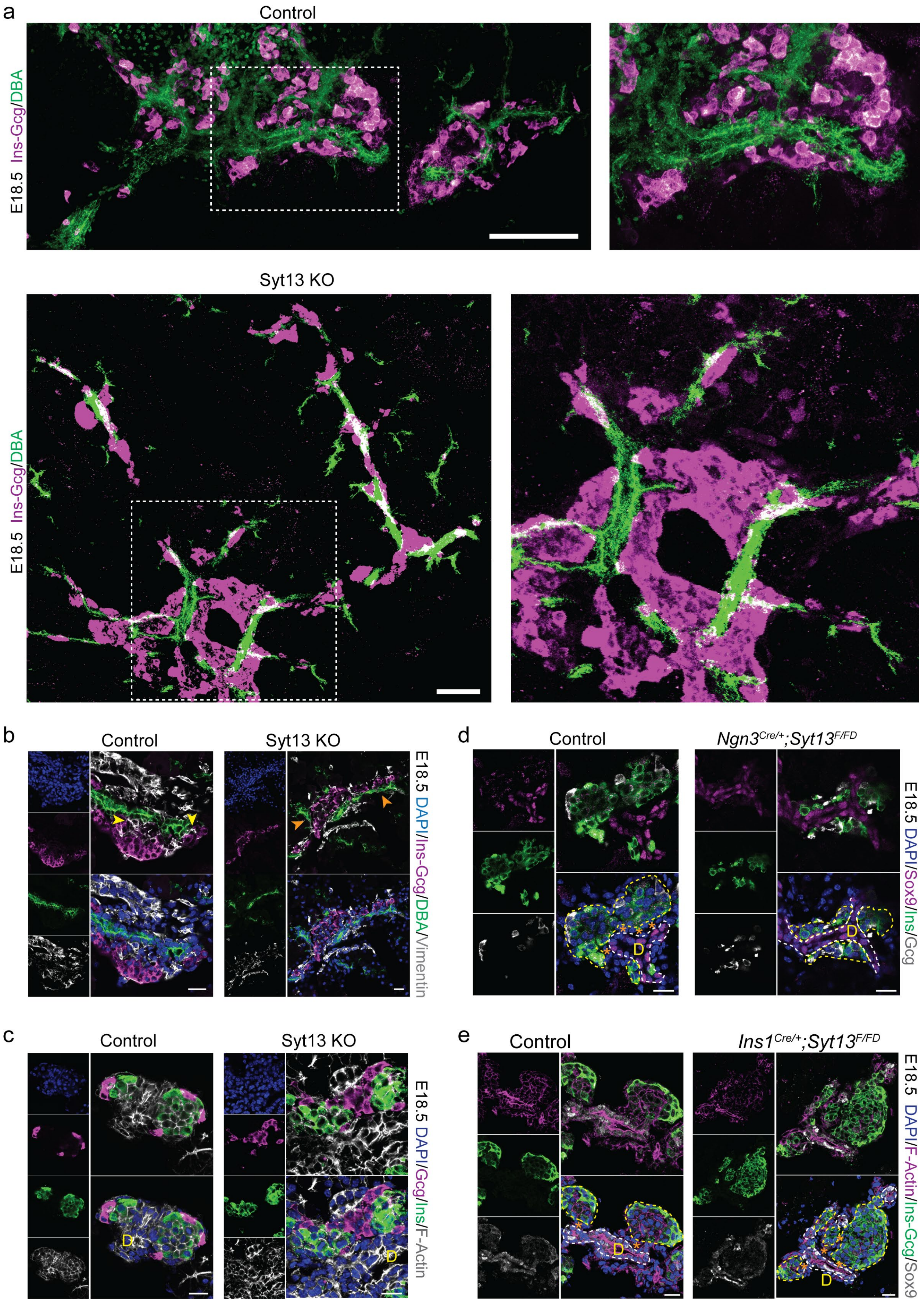
a
Control
E18.5 Ins-Gcg/DBA
Syt13 KO
E18.5 Ins-Gcg/DBA
b
Control
Syt13 KO
E18.5 DAPI/Ins-Gcg/DBA/Vimentin
c
Control
Syt13 KO
D
D
E18.5 DAPI/Gcg/Ins/F-Actin
d
Control
Ngn3^Cre/+;Syt13^F/FD
D
D
E18.5 DAPI/Sox9/Ins/Gcg
e
Control
Ins1^Cre/+;Syt13^F/FD
D
D
E18.5 DAPI/F-Actin/Ins-Gcg/Sox9

Supplementary Fig. 3. Syt13 deletion impairs proto-islet cluster positioning and arrangement. (a) Whole pancreata clearing and mount immunostaining. Pancreata were cleared using BABB and stained with insulin, glucagon and DBA (ductal marker). Representative pictures show the average of maximum projection of =<10 confocal Z-stack pictures. The 3D overview of these pictures is shown in Movies S1 and S2. (b) Staining of the pancreatic sections with mesenchymal marker, Vimentin. In the control, there are mesenchymal cells at the interface between proto-islets and epithelial cells (yellow arrowheads). In the Syt13 KO pancreata islets are more in direct connection with the epithelial cells (orange arrowheads). (c) Different α- and β-cells arrangement and positioning within the proto-islets in Syt13 KO compared to control. (d) Endocrine contact area with nearby epithelium in $Ngn3^{Cre/+}$; $Syt13^{F/FD}$ compared to control pancreatic sections. (e) Endocrine contact area with nearby epithelium in $Ins1^{Cre/+}$; $Syt13^{F/FD}$ and control pancreatic sections. D, duct. Yellow dashed lines indicate proto-islet clusters and white dashed lines indicate epithelium. Stars indicate space between endocrine and epithelial cells. Representative pictures from 4 independent pancreata (a-e). Scale bar 20 µm (b-d).

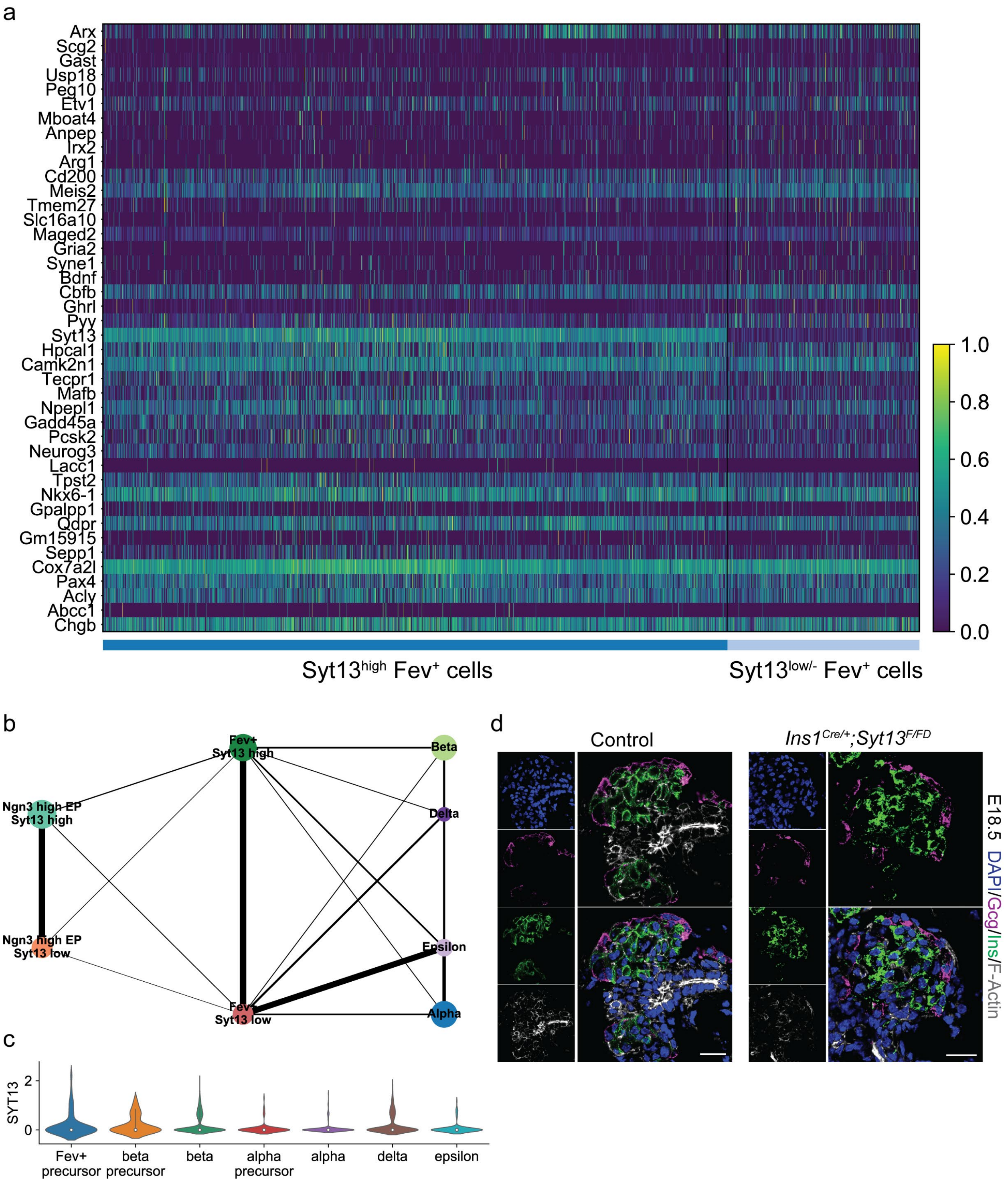

Supplementary Fig. 4. Increased levels of Syt13 in endocrine lineage link with β-cell fate. (a) Heatmap of top 20 differentially expressed genes in *Syt13*^{high} and *Syt13*$^{low/-}$ *Fev*$^{+}$ cells. (b) PAGA analysis corroborates the lineage relationship between *Syt13*^{high} and *Syt13*$^{low/-}$ precursors and *Fev*$^{+}$ cells with different types of

hormone$^+$ endocrine cells. (c) Violin plots showing *Syt13* expression in different endocrine lineages from human fetal pancreas. (d) IHC analysis of pancreatic sections from $Ins1^{Cre/+};Syt13^{F/FD}$ mice. Representative pictures from 3 independent pancreata. Scale bar 20 μm.

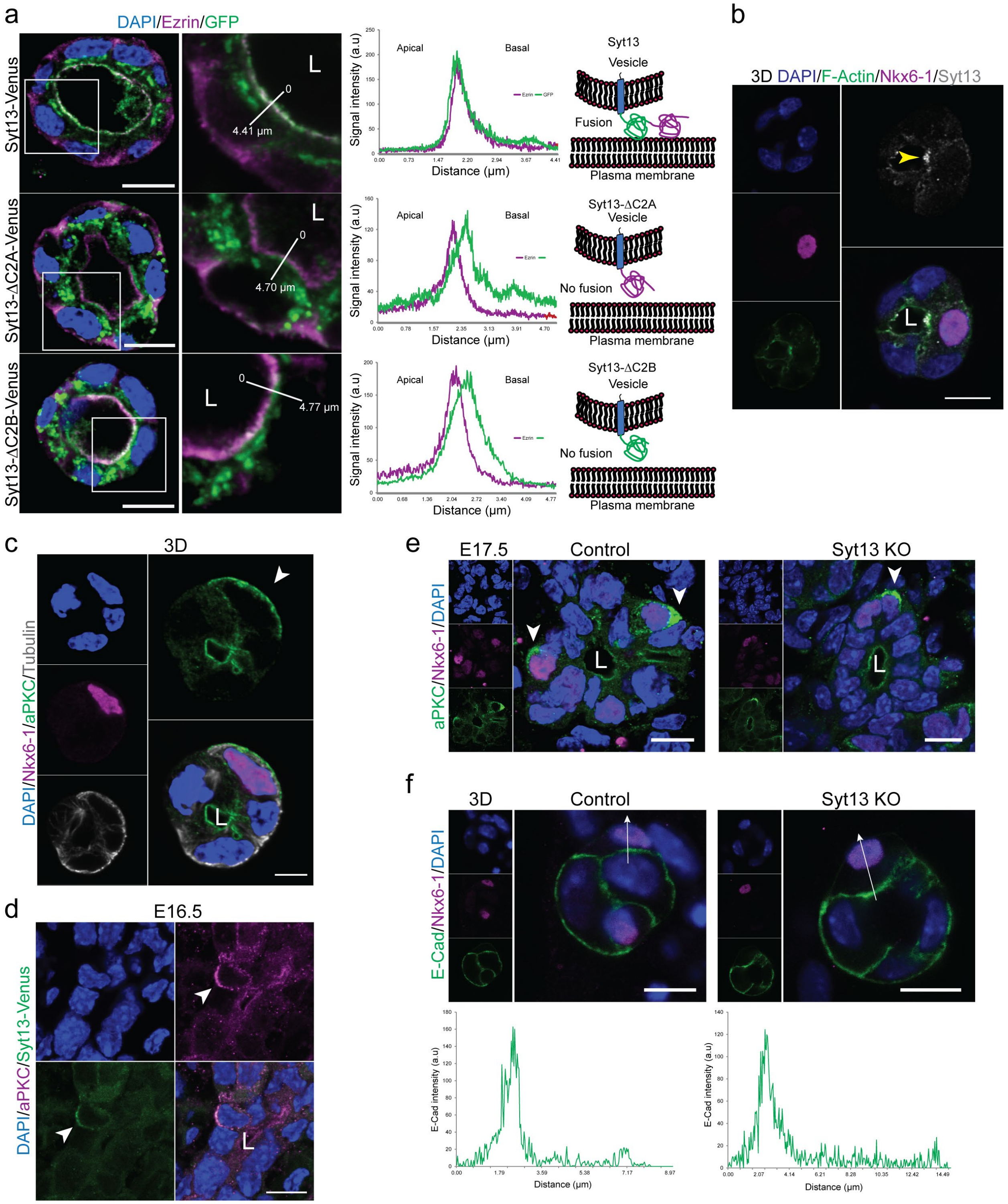

Supplementary Fig. 5. Syt13 polarized localization in epithelial and endocrine cells. (a) IF and plot profiles showing the accumulation of truncated Syt13 proteins under the apical domain in 3D MDCK

epithelial cysts, demonstrating that PM docking and fusion depends on the C2A and C2B domains. The scheme is created by the authors. (b) Syt13 protein and F-Actin colocalize (arrowhead) at the apical domain of differentiated endocrine cells residing within the pancreatic epithelium. (c) aPKC localizes at the front domain (arrowhead) of egressing endocrine cells in 3D epithelial cysts. (d) Pancreatic section indicating Syt13 and aPKC colocalization (arrowhead) at the front domain of endocrine cells. (e) Comparable levels of aPCK staining at the front domain (arrowheads) of endocrine cells in control and Syt13 KO pancreatic sections. (f) Similar E-Cadherin staining pattern (arrows) in control and Syt13-KO endocrine cells in the 3D culture. Representative pictures from 3 (a, c), 2 (b, d, f) and 3-4 (e) independent experiments. L, lumen. Scale bar 5 µm (c, e, f);10 µm (a, b, d).

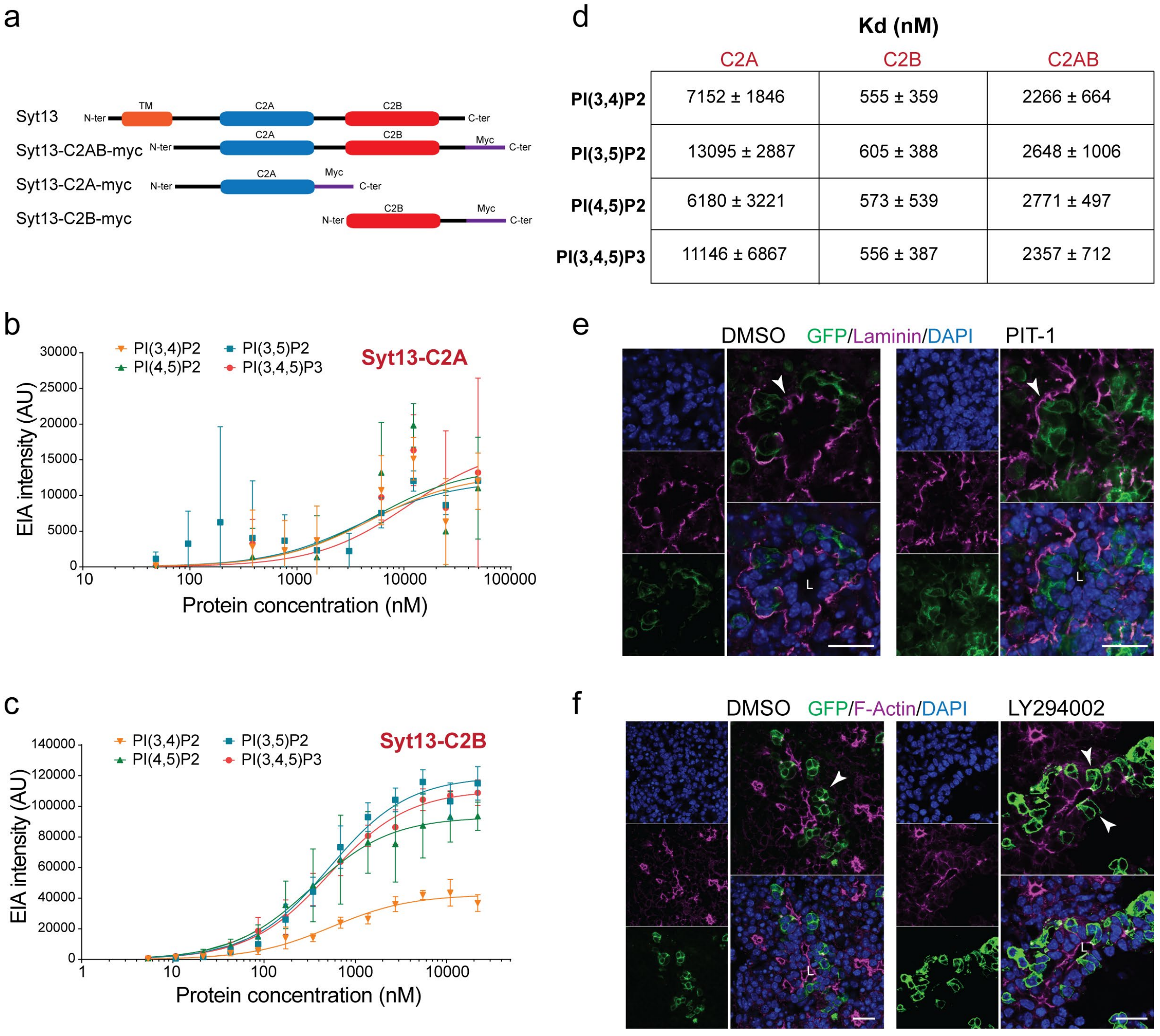

Kd (nM)

	C2A	C2B	C2AB
PI(3,4)P2	7152 ± 1846	555 ± 359	2266 ± 664
PI(3,5)P2	13095 ± 2887	605 ± 388	2648 ± 1006
PI(4,5)P2	6180 ± 3221	573 ± 539	2771 ± 497
PI(3,4,5)P3	11146 ± 6867	556 ± 387	2357 ± 712

Supplementary Fig. 6. Analysis of Syt13 lipid-binding properties. (a) Generation of different purified Syt13 protein variants for lipid-binding analysis. The scheme is created by the authors. (b) Binding of purified Syt13 C2A domain to 100 nm LUVs containing POPC/cholesterol/phosphoinositide 65/30/5 mol % followed by electrochemiluminescence-based immunoassay (EIA). n=3 independent experiments. (c) Binding of purified Syt13 C2B domain to LUVs containing POPC/cholesterol/phosphoinositide 65/30/5 mol % followed by liposomal electrochemiluminescence-based immunoassay (EIA). n=3 independent experiments. (d) Kd of different truncated Syt13 variants binding to LUVs containing POPC/cholesterol/phosphoinositide 65/30/5 mol %. (e) Treatment of E13.5 *Ngn3*$^{Cre/+}$; *ROSA26*$^{mTmG/mTmG}$

pancreata with PIP3 inhibitor (PIT-1). Arrowheads indicate basement membrane. (f) Treatment of explant culture of E13.5 *Ngn3*$^{Cre/+}$*; ROSA26*$^{mTmG/mTmG}$ pancreata with PI3K inhibitor LY294002 for 48 h impairs endocrine cell egression. Arrowheads indicate endocrine cells. L, lumen. Scale bar 20 μm (e, f). Representative pictures from 2 independent experiments (e, f). Data are represented as mean ± SD. Source data are provided as a Source Data file.

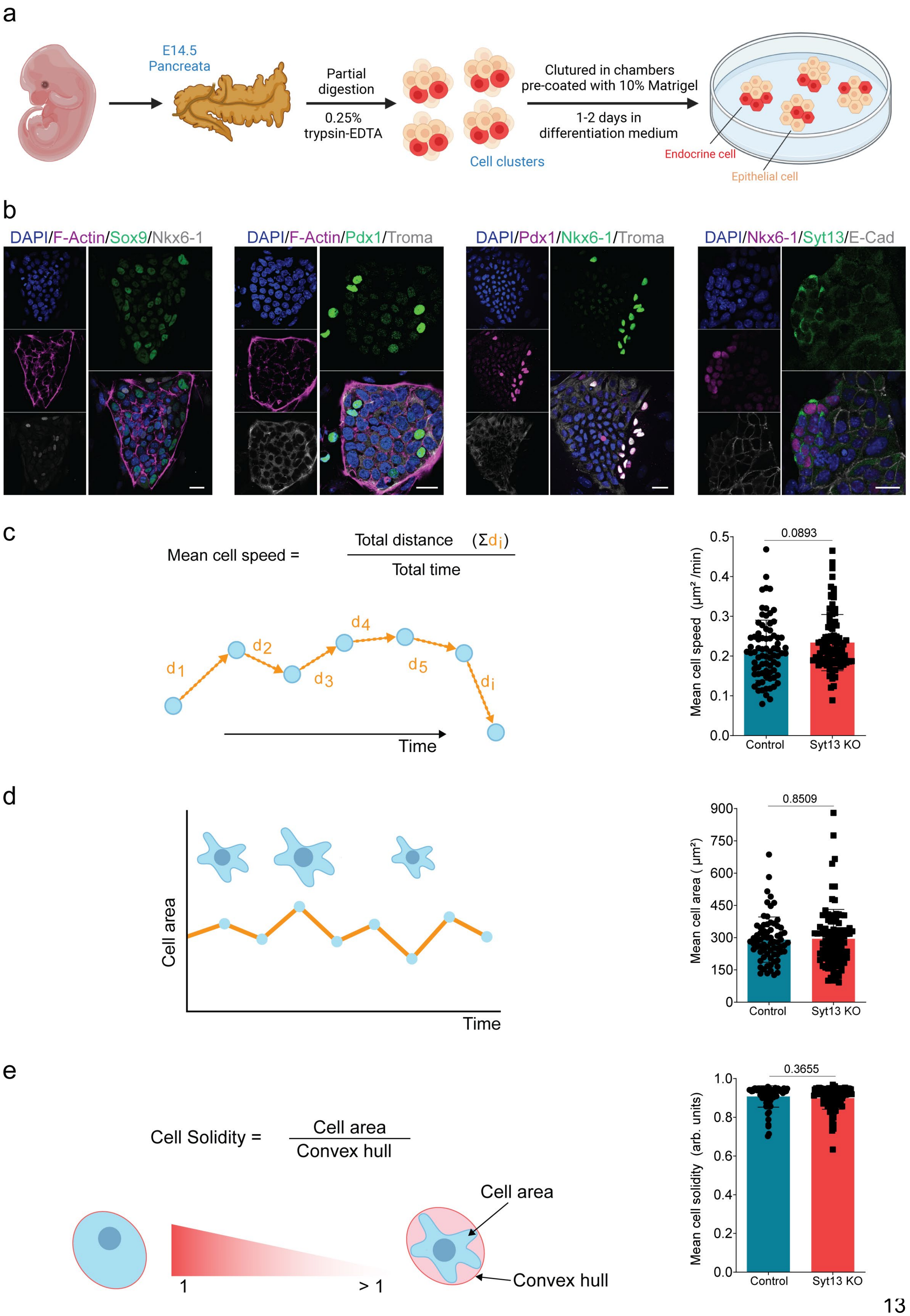
a
E14.5
Pancreata
Partial
digestion
0.25%
trypsin-EDTA
Cell clusters
Clutured in chambers
pre-coated with 10% Matrigel
1-2 days in
differentiation medium
Endocrine cell
Epithelial cell
b
DAPI/F-Actin/Sox9/Nkx6-1
DAPI/F-Actin/Pdx1/Troma
DAPI/Pdx1/Nkx6-1/Troma
DAPI/Nkx6-1/Syt13/E-Cad
c
Mean cell speed = Total distance (Σdi) / Total time
d1
d2
d3
d4
d5
di
Time
Mean cell speed (µm² /min)
0.0893
Control
Syt13 KO
d
Cell area
Time
Mean cell area (µm²)
0.8509
Control
Syt13 KO
e
Cell Solidity = Cell area / Convex hull
1
> 1
Cell area
Convex hull
Mean cell solidity (arb. units)
0.3655
Control
Syt13 KO

Supplementary Fig. 7. Analysis of endocrine cell dynamics in vitro. (a) Scheme of the procedure to culture primary pancreatic cells in a 2D condition. Created with BioRender.com. (b) Characterization of the 2D culture of mouse embryonic pancreatic epithelial cells. Scale bar 20 μm. Representative pictures from 2 independent experiments. (c-e) Means of speed, area and solidity of Syt13 KO and control endocrine cells. These analyses were performed using the time-lapse imaging data represented in Movies S3, 4. The schemes are created by the authors. (c) n=77 control and 96 KO endocrine cells from 4 different pancreata; (d and e) n=71 control and 96 KO endocrine cells from 4 different pancreata. Two-sided t-test. Data are represented as mean ± SD. Source data are provided as a Source Data file.

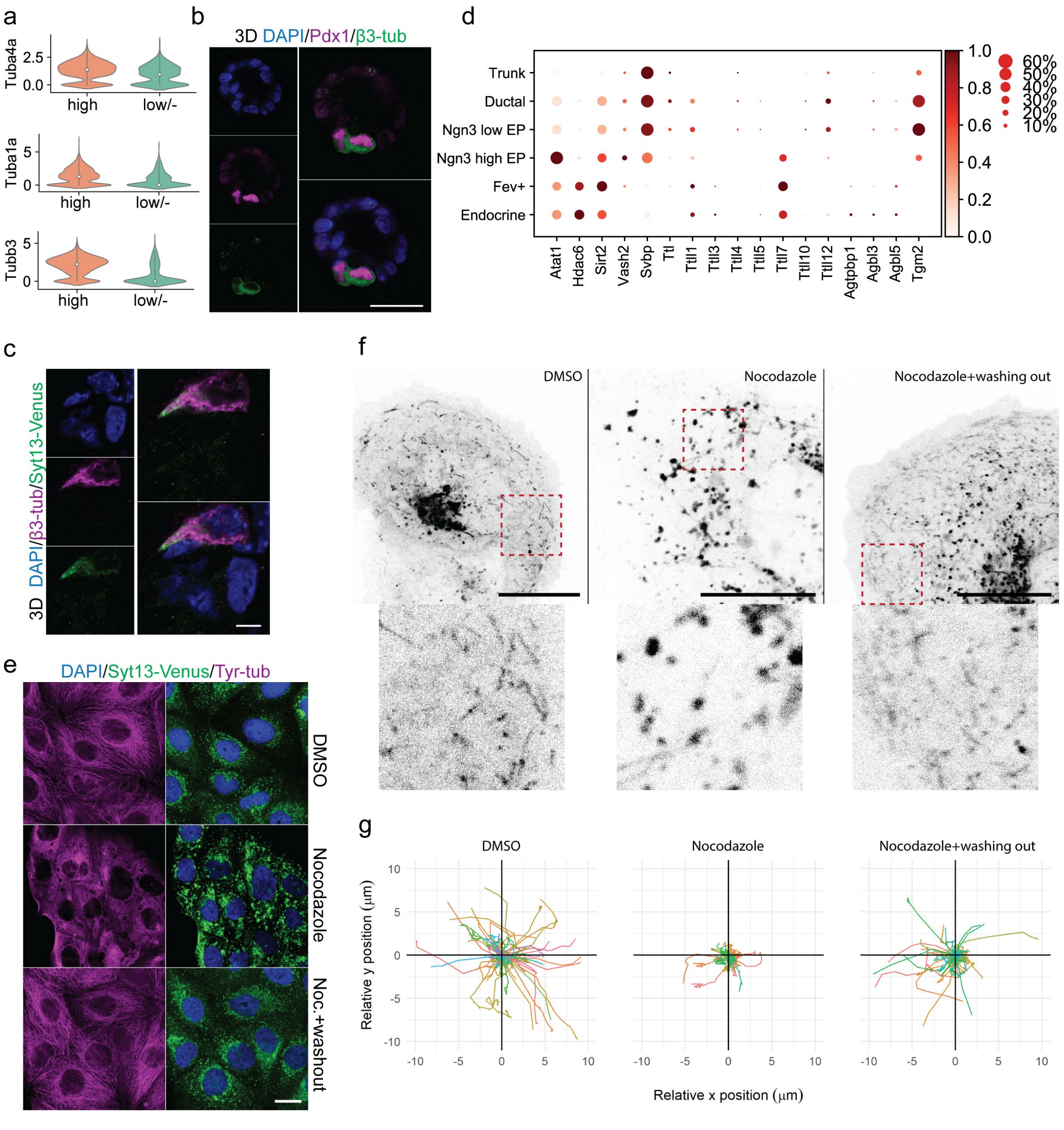

Supplementary Fig. 8. Syt13 associates with MT cytoskeleton. (a) Violin plots of the expression levels of several tubulin-encoding genes in *Syt13*^{high} and *Syt13*$^{low/-}$ precursors. (b) Increased expression of β3-tubulin during endocrinogenesis. (c) IF analysis shows co-expression of Syt13 with β3-tubulin in endocrine lineage. (d) Dot plots of the expression levels of genes encoding enzymes involved in tubulin post-translational modification during endocrinogenesis. (e) IF showing disruption of MT network in MDCK

cells upon administration of nocodazole. (f) Represented pictures of intracellular vesicles in Syt13-Venus overexpressing MDCK cells obtained from time-lapse imaging. The Venus signals are shown in gray. The lower panel magnifies the endosomal compartments. (g) Representative scheme of movement distance of the intracellular vesicles in MDCK overexpressing Syt13-Venus. For simplicity, the data from one biological replicate are shown. Scale bar 10 µm (c); 20 µm (b, e, f). Representative pictures from 2 (b, c, e) and 3 (f) independent experiments.

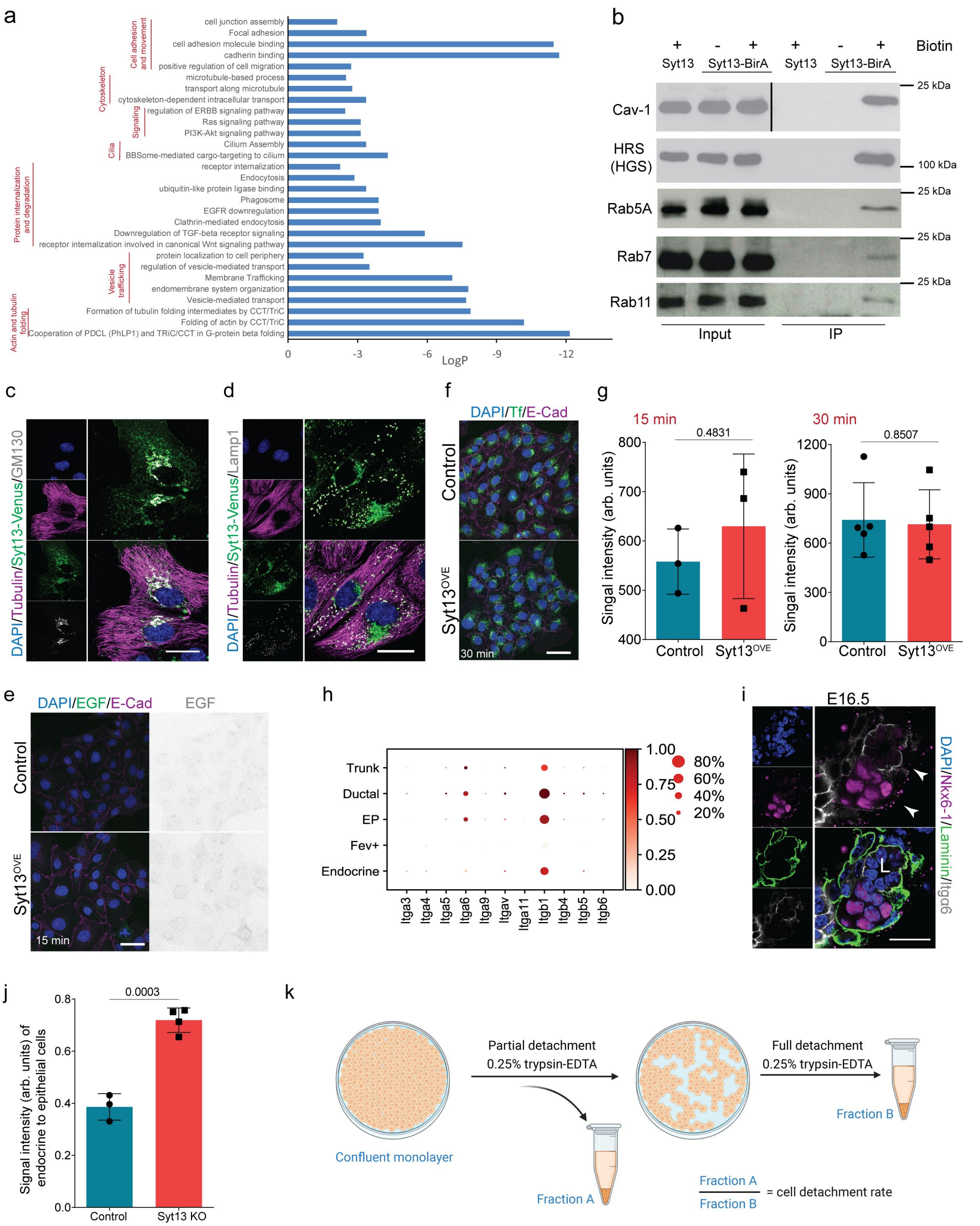

a
Cell adhesion and movement
cell junction assembly
Focal adhesion
cell adhesion molecule binding
cadherin binding
positive regulation of cell migration
Cytoskeleton
microtubule-based process
transport along microtubule
cytoskeleton-dependent intracellular transport
Signaling
regulation of ERBB signaling pathway
Ras signaling pathway
PI3K-Akt signaling pathway
Cilia
Cilium Assembly
BBSome-mediated cargo-targeting to cilium
Protein internalization and degradation
receptor internalization
Endocytosis
ubiquitin-like protein ligase binding
Phagosome
EGFR downregulation
Clathrin-mediated endocytosis
Downregulation of TGF-beta receptor signaling
receptor internalization involved in canonical Wnt signaling pathway
Vesicle trafficking
protein localization to cell periphery
regulation of vesicle-mediated transport
Membrane Trafficking
endomembrane system organization
Vesicle-mediated transport
Actin and tubulin folding
Formation of tubulin folding intermediates by CCT/TriC
Folding of actin by CCT/TriC
Cooperation of PDCL (PhLP1) and TRiC/CCT in G-protein beta folding
0
-3
-6
-9
-12
LogP
b
+ - + + - +
Biotin
Syt13
Syt13-BirA
Syt13
Syt13-BirA
Cav-1
HRS (HGS)
Rab5A
Rab7
Rab11
25 kDa
100 kDa
25 kDa
25 kDa
25 kDa
Input
IP
c
DAPI/Tubulin/Syt13-Venus/GM130
d
DAPI/Tubulin/Syt13-Venus/Lamp1
f
DAPI/Tf/E-Cad
Control
Syt13OVE
30 min
g
15 min
0.4831
Singal intensity (arb. units)
800
700
600
500
400
Control
Syt13OVE
30 min
0.8507
Singal intensity (arb. units)
1200
900
600
300
0
Control
Syt13OVE
e
DAPI/EGF/E-Cad
EGF
Control
Syt13OVE
15 min
h
Trunk
Ductal
EP
Fev+
Endocrine
Itga3
Itga4
Itga5
Itga6
Itga9
Itgav
Itga11
Itgb1
Itgb4
Itgb5
Itgb6
1.00
0.75
0.50
0.25
0.00
80%
60%
40%
20%
i
E16.5
DAPI/Nkx6-1/Laminin/Itga6
L
j
0.0003
Signal intensity (arb. units) of endocrine to epithelial cells
0.8
0.6
0.4
0.2
0.0
Control
Syt13 KO
k
Partial detachment
0.25% trypsin-EDTA
Full detachment
0.25% trypsin-EDTA
Confluent monolayer
Fraction A
Fraction B
Fraction A
Fraction B
= cell detachment rate

Supplementary Fig. 9. Syt13 functions as a trafficking protein. (a) Selected terms of pathway analysis of Syt13 interactome partners identified by BioID approach. (b) IF followed by western blot analysis indicates the close proximity of Syt13 with several proteins involved in endocytosis and vesicle trafficking in MDCK cells. (c, d) Colocalization of overexpressed Syt13 with GM130 and Lamp1 in MDCK cells. (e) IF of EGF uptake in control and Syt13^{OVE} MDCK cells after 15 min incubation. (f, g) IF and quantification of labeled transferrin uptake in control and Syt13^{OVE} MDCK cells after 15- and 30-min incubation. n=3 (15 min) and n=5 (30 min) independent experiments. Two-sided t-test. (h) Dot plot shows the expression levels of genes encoding different integrin subunits during mouse endocrinogenesis. (i) Reduction of Itgα6 (arrowheads) but not laminin in endocrine clusters compared to the ductal epithelial cells stained in pancreatic sections at E16.5. (j) Quantification of Itgβ4 at the basal domain of endocrine cells compared to the nearby epithelial cells. n=3 (control) and n=4 (KO) pancreata. Two-sided t-test. (k) Scheme of the procedure of the detachment assay in MDCK cells. Fraction A and B are cell numbers. Created with BioRender.com. Scale bar 20 µm (c, d, i); 50 µm (e, f). Representative pictures from 2 (b, c, d, i), 3 (e) and 5 (f) independent experiments. Two-sided t-test. Data are represented as mean ± SD. Source data are provided as a Source Data file.

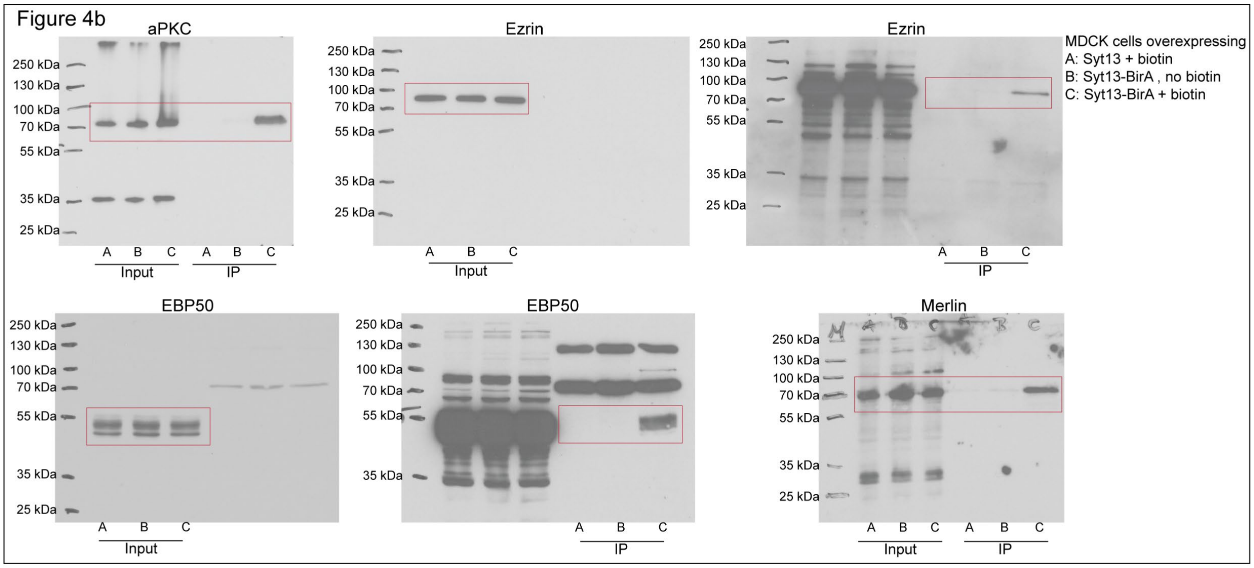
Figure 4b
aPKC
Ezrin
Ezrin
MDCK cells overexpressing
A: Syt13 + biotin
B: Syt13-BirA , no biotin
C: Syt13-BirA + biotin
EBP50
EBP50
Merlin
250 kDa
130 kDa
100 kDa
70 kDa
55 kDa
35 kDa
25 kDa
A B C A B C
Input
IP

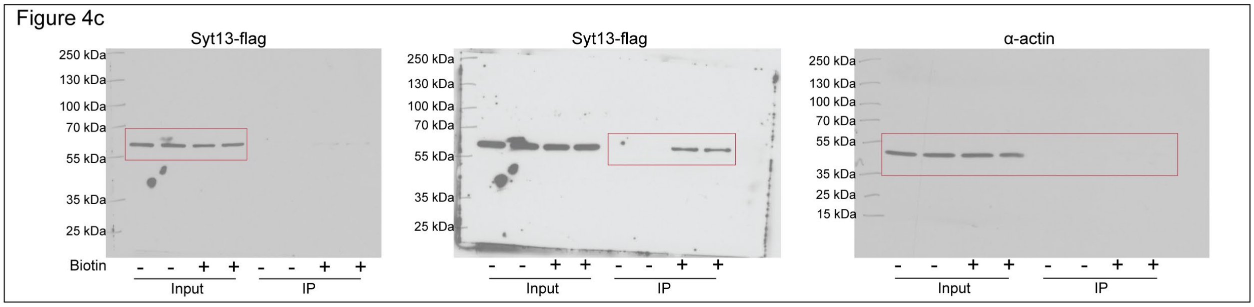
Figure 4c
Syt13-flag
Syt13-flag
α-actin
250 kDa
130 kDa
100 kDa
70 kDa
55 kDa
35 kDa
25 kDa
15 kDa
Biotin
- - + + - - + +
Input
IP

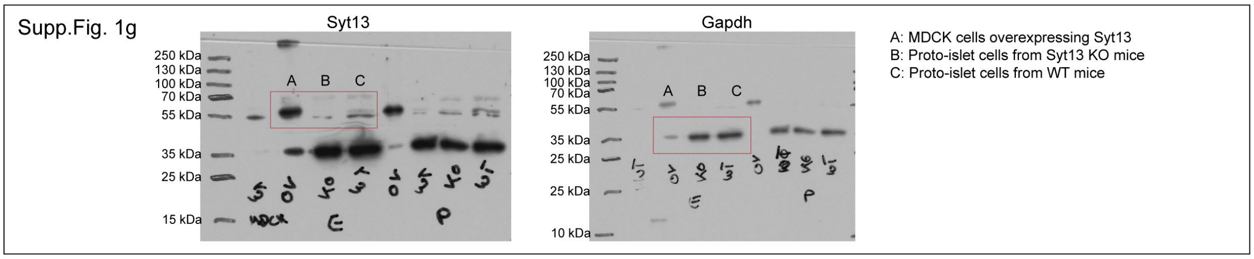
Supp.Fig. 1g
Syt13
Gapdh
A B C
250 kDa
130 kDa
100 kDa
70 kDa
55 kDa
35 kDa
25 kDa
15 kDa
10 kDa
A: MDCK cells overexpressing Syt13
B: Proto-islet cells from Syt13 KO mice
C: Proto-islet cells from WT mice

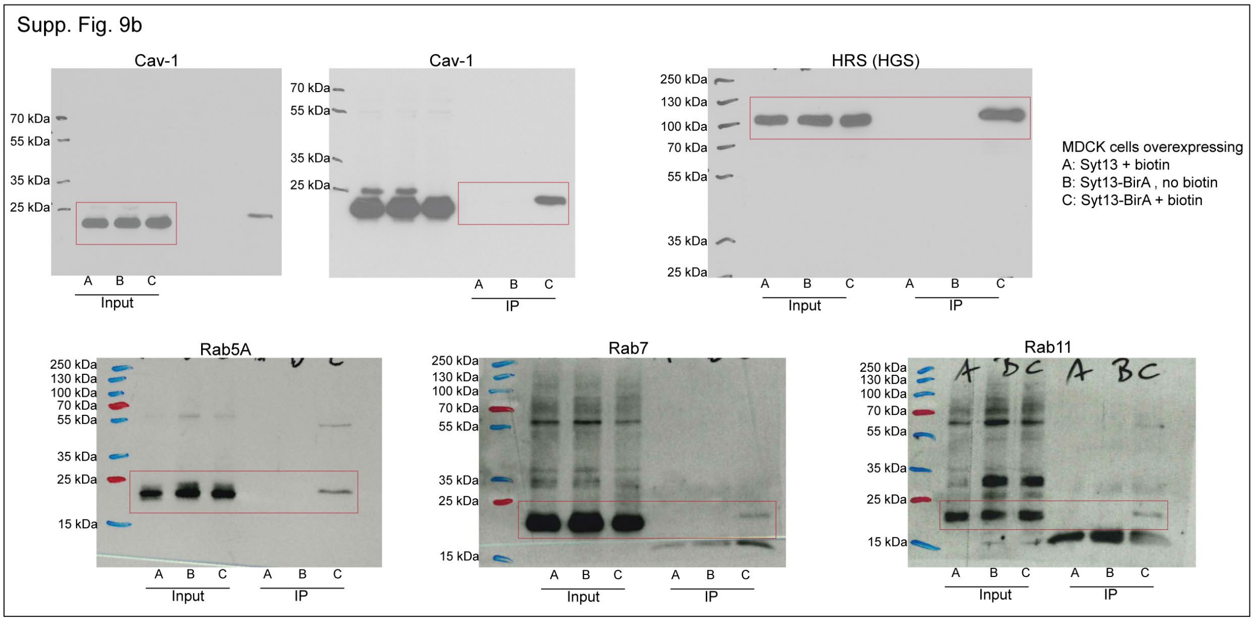
Supp. Fig. 9b
Cav-1
Cav-1
HRS (HGS)
MDCK cells overexpressing
A: Syt13 + biotin
B: Syt13-BirA , no biotin
C: Syt13-BirA + biotin
Rab5A
Rab7
Rab11
250 kDa
130 kDa
100 kDa
70 kDa
55 kDa
35 kDa
25 kDa
15 kDa
A B C A B C
Input
IP

Supplementary Fig. 10. The uncropped and unprocessed scans of all the shown western blots. The used bands are indicated within the red boxes.
