# Supplementary material for: Synaptotagmin-13 orchestrates pancreatic endocrine cell egression and islet morphogenesis
Source: Nat Commun. 2022 Aug 4;13:4540. doi: 10.1038/s41467-022-31862-8 (PMC9352765; doi:10.1038/s41467-022-31862-8)
Supplement: Supplementary file 2 — Description of Additional Supplementary Files [file 41467_2022_31862_MOESM2_ESM.pdf]

Description of Additional Supplementary Files

File Name: Supplementary Data 1
Description: Detailed information of genotyping primers, generated plasmids, used primary and secondary antibodies and used qPCR probs.

File Name: Supplementary Data 2
Description: Differential expressed genes in *Syt13*^{low} and *Syt13*^{high} endocrine precursors and *Fev*$^{+}$ cells and the corresponding pathway analyses. For statistical analysis, two-sided Welch's t-test was used. Benjamini-Hochberg correction was applied for multiple testing.

File Name: Supplementary Data 3
Description: List of potential Syt13 interaction partner proteins identified by BioID proximity labeling and the corresponding pathway analysis.

File Name: Supplementary Movie 1
Description: 3D reconstruction of whole-mount imaged control pancreata at E18.5.

File Name: Supplementary Movie 2
Description: 3D reconstruction of whole-mount imaged Syt13 KO pancreata at E18.5.

File Name: Supplementary Movie 3
Description: Time-lapse imaging of endocrine cell dynamics from WT pancreata in a 2D culture system.

File Name: Supplementary Movie 4
Description: Time-lapse imaging of endocrine cell dynamics from Syt13 KO pancreata in a 2D culture system.

File Name: Supplementary Movie 5
Description: Time-lapse imaging of Syt13-positive vesicle movement in MDCK cells.

File Name: Supplementary Movie 6
Description: Time-lapse imaging showing colocalization of Syt13-positive vesicles with lysosomes (LysoTracker) in MDCK cells.
