# Supplementary material for: Synaptotagmin-13 orchestrates pancreatic endocrine cell egression and islet morphogenesis
Source: Nat Commun. 2022 Aug 4;13:4540. doi: 10.1038/s41467-022-31862-8 (PMC9352765; doi:10.1038/s41467-022-31862-8)
Supplement: Supplementary file 12 — Reporting Summary [file 41467_2022_31862_MOESM12_ESM.pdf]

nature portfolio

Corresponding author(s): Heiko Lickert and Mostafa Bakhti

Last updated by author(s): 2022.06.08

Reporting Summary

Nature Portfolio wishes to improve the reproducibility of the work that we publish. This form provides structure for consistency and transparency in reporting. For further information on Nature Portfolio policies, see our Editorial Policies and the Editorial Policy Checklist.

Statistics

For all statistical analyses, confirm that the following items are present in the figure legend, table legend, main text, or Methods section.

n/a	Confirmed	
☐	☒	The exact sample size (*n*) for each experimental group/condition, given as a discrete number and unit of measurement
☐	☒	A statement on whether measurements were taken from distinct samples or whether the same sample was measured repeatedly
☐	☒	The statistical test(s) used AND whether they are one- or two-sided<br>*Only common tests should be described solely by name; describe more complex techniques in the Methods section.*
☒	☐	A description of all covariates tested
☐	☒	A description of any assumptions or corrections, such as tests of normality and adjustment for multiple comparisons
☐	☒	A full description of the statistical parameters including central tendency (e.g. means) or other basic estimates (e.g. regression coefficient) AND variation (e.g. standard deviation) or associated estimates of uncertainty (e.g. confidence intervals)
☐	☒	For null hypothesis testing, the test statistic (e.g. *F*, *t*, *r*) with confidence intervals, effect sizes, degrees of freedom and *P* value noted<br>*Give P values as exact values whenever suitable.*
☒	☐	For Bayesian analysis, information on the choice of priors and Markov chain Monte Carlo settings
☒	☐	For hierarchical and complex designs, identification of the appropriate level for tests and full reporting of outcomes
☒	☐	Estimates of effect sizes (e.g. Cohen's *d*, Pearson's *r*), indicating how they were calculated

Our web collection on statistics for biologists contains articles on many of the points above.

Software and code

Policy information about availability of computer code

Data collection	Immunostaining: The stained tissues and cells were scanned with Leica or Zeiss confocal microscope. All images were obtained with a Leica microscope of the type DMI 6000 using the LAS AF software and Zeiss LSM880 inverted confocal microscope.<br><br>Western blot: The signals were detected by enhanced chemiluminescence (Thermo Scientific).<br><br>The qPCR was performed using Viia7 (Thermo Fisher Scientific).<br><br>Cells isolation was done using an Aria III (BD Biosciences, Heidelberg, Germany).<br><br>LC-MS/MS analysis was performed on Ultimate3000 nanoRSLC systems (Thermo Scientific) coupled to an Orbitrap Fusion Tribrid mass spectrometer (Thermo Scientific) by a nanospray ion source.<br><br>For assessment of lipid-binding properties of Syt13 purified protein, the readout was performed on a Meso Scale Discovery SECTOR Imager 6000 chemiluminescence reader.
Data analysis	Confocal images were processed, analyzed and quantified by Leica LAS-AF (Version 2.7.7.12402), LAS-X (Version 3.5.6), Zen 2.3 SP1 FP3 (black) 64bit, ImageJ (1.53c (Fiji) and IMARIS 9.3, RStudio 2021.09.0+351 version softwares.<br><br>All statistics, unless specified were performed using Graphpad Prism software 9 version 9.0.1 (GraphPad Software Inc., La Jolla, CA).<br><br>MS/MS data were analyzed using the MaxQuant software (version 1.6.1.0).<br><br>scRNAseq data were analyzed using python 3.7.6. Processing and analyses of publicly available count matrices were carried out using custom

scripts run as jupyter notebooks (jupyterlab v2.1.3), the scRNA-seq analysis suite scanpy v1.4.4 and the following python packages: anndata v0.7.1, umap v0.3.10, numpy v1.17.0, scipy v1.3.1, pandas v0.25.1, scikit-learn v0.21.3, statsmodels v0.10.1, python-igraph v0.7.1, louvain v0.6.1, seaborn v0.11.1, matplotlib v3.0.3, xlsxwriter v1.3.7, rpy2 v3.3.5, anndata2ri v1.0.4, gseapy v0.10.2. All jupyter notebooks with the script are deposited in a repository on github: https://github.com/theislab/Bakthi2021_pancreas_Syt13.

For manuscripts utilizing custom algorithms or software that are central to the research but not yet described in published literature, software must be made available to editors and reviewers. We strongly encourage code deposition in a community repository (e.g. GitHub). See the Nature Portfolio guidelines for submitting code & software for further information.

Data

Policy information about availability of data

All manuscripts must include a data availability statement. This statement should provide the following information, where applicable:

- Accession codes, unique identifiers, or web links for publicly available datasets
- A description of any restrictions on data availability
- For clinical datasets or third party data, please ensure that the statement adheres to our policy

The mass spectrometry proteomics data have been deposited to the ProteomeXchange Consortium via the PRIDE partner repository with the dataset identifier PXD026699.

Custom code for all analyses of scRNA-seq data are available as jupyter notebooks in a github repository: https://github.com/theislab/2022_Bakhti_pancreas_Syt1361. All data can be downloaded from the sources indicated in the notebooks as well as the methods part.

Processed and normalized scRNA-seq count matrix and cell annotations of mouse embryonic pancreatic cells (Bastidas-Ponce et al., 2019) were downloaded from GEO (accession number: GSE132188).

Processed and normalized scRNA-seq count matrix and cell annotations of human in vitro stem cell differentiation (Veres et al., 2019) were downloaded from GEO (accession number GSE114412).

Raw scRNA-seq count matrix from human fetal pancreas (Cao et al. 2020) were downloaded from the data visualization center descartes (https://descartes.brotmanbaty.org/bbi/human-gene-expression-during-development/).

Field-specific reporting

Please select the one below that is the best fit for your research. If you are not sure, read the appropriate sections before making your selection.

☒ Life sciences ☐ Behavioural & social sciences ☐ Ecological, evolutionary & environmental sciences

For a reference copy of the document with all sections, see nature.com/documents/nr-reporting-summary-flat.pdf

Life sciences study design

All studies must disclose on these points even when the disclosure is negative.

Sample size	No statistical method was used to predetermine sample size. A minimum of 3 independent experiments were carried out for statistical analysis based on similar reported studies. When possible more samples were used. Sample numbers are indicated in the figure legends.
Data exclusions	There were no data exclusions in this study.
Replication	All attempts for data replication in in vivo and in vitro conditions were successful with independents biological replicates. Exact replicate numbers are indicated in the figure legends.
Randomization	For mouse studies, group were allocated based on their genotype. All in vitro experiments were allocated randomly. The plate wells for seeding cells from different conditions were selected randomly. For microscopy, random fields of view were chosen for imaging and analysis.
Blinding	Quantification of confocal images were performed blindly. For the collection of tissues investigators were not blinded, as it was necessary to have the genotype information of offspring due to the crossing of heterozygous parents. For the other experiments, when the experiments were performed by more than two people it was blinded. This was not possible when the whole experiment was done by a single person.

Reporting for specific materials, systems and methods

We require information from authors about some types of materials, experimental systems and methods used in many studies. Here, indicate whether each material, system or method listed is relevant to your study. If you are not sure if a list item applies to your research, read the appropriate section before selecting a response.

Materials & experimental systems

n/a	Involved in the study
☐	☒ Antibodies
☐	☒ Eukaryotic cell lines
☒	☐ Palaeontology and archaeology
☐	☒ Animals and other organisms
☒	☐ Human research participants
☒	☐ Clinical data
☒	☐ Dual use research of concern

Methods

n/a	Involved in the study
☒	☐ ChIP-seq
☒	☐ Flow cytometry
☒	☐ MRI-based neuroimaging

Antibodies

Antibodies used

List of primary antibodies

Mouse anti-Actin (BD #612656), Clone C4, Carim et al., Curr Biol. 2019; Hegde et al., Elife 2019.
Mouse anti-Tubulin-alpha (Sigma, #T6199), Clone DM1A, Dutcher, S.K., et al., Curr. Opin. Cell Biol. 2001.
Mouse anti-Tubulin-Acetylated (Sigma, #T7451), Clone 6-11B-1, Morales and Fifkova, Cell Tissue Res. 1991.
Rabbit anti-Flag S(igma, #F7425), Xiao et al. Cell death and differentiation, 2020.
Chicken anti-GFP (Aves Labs, #GFP-1020), Zhenyu et al., Stem Cell Reports 2018.
Rabbit anti-FOXA2 (HNF-3β) (Cell signaling, #8186S), Clone D56D6, Bastidas-Ponce et al., Mol Metab 2017.
Rat anti-Somatostatin (Invitrogen, #MA5-16987), Clone YC7, Parajuli et al., Life Sci Alliance 2020.
Mouse anti-c-Myc clone 9E10 (Sigma, #M4439), Del Barrio et al. Development 2007.
Mouse anti-Ezrin (clone 3C12) (Sigma, #E8897), Veland et al. PloS one 2013.
Rabbit anti-PDX1 (D59H3) (Cell Signaling, #5679), Bastidas-Ponce et al., Mol Metab 2017.
Goat anti-NKX6.1 (R&D systems, #AF5857), Burtscher et al., Int J Mol Sci. 2021.
Rat anti-CD49f (BD, #555734), Clone GoH3 (RUO), Bakthi et al., Mol Metab 2019.
Rabbit anti-SOX9 (Millipore, #AB5535), Saha et al. Nature 2014.
Rat anti-Tubulin clone YL1/2 (Tyr-Tub) (Millipore, #MAB1864), Nakaya et al. Nat. Cell Biol. 2008.
Goat anti-Glucagon (Bio-Rad, #4660-1140), Li et al., BMC Dev Biol. 2010.
Guinea Pig anti-Glucagon 8TAKARA, #M182), Sachs et al., Nat Metab 2020.
Rabbit anti-SYT13 (Abcam, #ab154695)
Rabbit anti-c-Myc (Sigma, #C3956), Huasheng Yu et al. eLife 2019.
Rat anti-Cadherin - E (DECMA-1) (Kremmer, #SC-59778), Bakthi et al., Mol Metab. 2019.
Rabbit anti-PKC ζ (Santa Cruz, #sc-21617), Bakthi et al., Mol Metab 2019.
Rabbit anti-Insulin (C27C9) (Cell signaling, #3014), Sachs et al., Nat Metab 2020.
Rabbit anti-EBP50/NHERF1 (Abcam, #ab3452), Bakthi et al., Mol Metab. 2019.
Mouse anti-NF2 / Merlin (Abcam, #ab88957), Clone AF1G4, Zhou et al., J Cell Physiol., 2022.
Rabbit anti-Laminin (Sigma, #L9393), Ucci et al. Int J Mol Sci. 2019.
Rabbit anti-HRS (D7T5N) (HGS) (Cell signaling, #15087), Clone D7T5N, Tan et al. EMBO J 2015.
Rat anti-Integrin beta 4 (Abcam, #ab25254), Clone 346-11A, Kato et al., Sci Rep. 2019.
Goat anti-Pdx1 (R&D Systems, #AF2419), Liu et al., Nature Communications 2021.
Mouse anti-ParD6B (Santa Cruz, #sc-166405), Clone B-10, Zhu et al., Elife 2021.
Rabbit anti-Chromogranin A (Chr-A) (Immunostar, #20086), He et al., Cell Rep. 2021.
Rabbit anti-Tubulin beta III (Abcam, #ab18207), Zhang et al. FEBS Open Bio 2021.
Rabbit anti-Caveolin-1 (D46G3) (Cell signaling, #3267T), Xuemeng Shi, et. al. J Mol Cell Biol 2022.
Rabbit anti-Rab5A (E6N8S) (Cell signaling, #46449T), Kaneshiro, et. al. iScience 2022.
Rabbit anti-Rab7 (D95F2) (Cell signaling, #9367T), Yang Liu, et. al. Front Genet 2022.
Rabbit anti-Rab11 (D4F5) (Cell signaling, #5589T), Clément Auger, et. al. Int J Mol Sci 2022.
Mouse anti-LAMP1 (BIOMOL, #VAM-EN001), Clone H4A3, Dai et al., Nat Commun 2019.
Goat anti-Collagen type IV (Millipore, #AB769), Daley et al., Dev Dyn 2011.
Mouse anti-Tubulin-alpha-Glu (Synaptic system, #302011), Clone 1D5,Van der Heiden et al., Dev Dyn. 2006.
Rat anti-KRT8 (TROMA-I) (Hybridoma, Kremmer), Strunz et al., Nat Commun 2020.
Mouse anti-GM130 (BD, #610822), Clone 35/GM130 (RUO), Marra et al., Nat Cell Biol. 2001.
Rabbit anti-EEA1 (NEB, #2411S), Fernbach et al.,Cell Rep. 2022.
Rabbit anti-Vimentin (abcam, #ab92547), Clone EPR3776, Wu et al., Clin Hemorheol Microcirc. 2021.
Rabbit anti-Paxillin (abcam, #ab32084), Clone Y113, Kilinc et al., Mol Biol Cell. 2021.

List of secondary antibodies

Anti-Chicken Cy™2 (Dianova, #703-225-155)
Anti-Rat Alexa Flour® 488 (Life Technologies, #A-21208)
Anti-Rat Cy™3 (Dianova, #712-165-153)
Anti-Rat Alexa Flour® 647 (Dianova, #712-605-150)
Anti-Rabbit Alexa Flour® 488 (Invitrogen, #A11055)
Anti-Rabbit Alexa Flour® 555 (Invitrogen, #A21206)
Anti-Rabbit Alexa Flour® 647 (Dianova, #A31573)
Anti-Guinea pig Alexa Flour® 488 (Dianova, #706-545-148)
Anti-Guinea pig Cy™3 (Dianova/Jackson, #706-165-148)
Anti-Guinea pig Alexa Flour® 649 (Dianova, #706-495-148)

Anti-Goat Alexa Flour® 555 (Invitrogen, #A21432)
Anti-Goat Alexa Flour® 633 (InvitrogenvA21082)
Anti-Mouse Alexa Flour® 488 (Invitrogen, #A21202)
Anti-Mouse Alexa Flour® 555 (Invitrogen, #A31570)
Phalloidin Alexa Flour® 546 (Invitrogen, #A22283)
SiR-Actin Alexa Flour® 647 (Cytoskeleton, Inc., #CY-SC001)
Anti-Mouse Cy™5 (Dianova, #715-175-151)
Anti-Mouse IgG (H+L), HRP (Dianova/Jackson, #DAB-087641)
Anti-Rabbit IgG (H+L), HRP (Dianova/Jackson, #DAB-087729)
Anti-Mouse IgG (H+L), HRP (Dianova/Jackson, #115-035-146)
Anti-Rabbit IgG (H+L), HRP (Dianova/Jackson, #111-035-144)
Anti-Mouse IgG (H+L), HRP (Cell signaling, #7076P2)
Anti-Rabbit IgG (H+L), HRP (Cell signaling, #7074P2)
Biotin Streptavidin, HRP- (Conjugated Life Technologies, #21126)
Dolichos Biflorus Agglutinin (DBA), Alexa Fluor 488 (Vector Laboratories, #FL-1031)

Validation

All antibodies were validated based on published research articles. The references are included in the Supplementary table 1 and in the section above.

Syt13 antibody was validated using Syt13 overexpression in cell lines and immunostaining and Western blotting of tissues from Syt13 knockout mice.

Eukaryotic cell lines

Policy information about cell lines

Cell line source(s)

MDCK cells (NBL-2) were purchased from Sigma (#85011435).

MDCK cells over-expressing different variants of Syt13 proteins were generated in this study.

Authentication

Cell lines were not authenticated.

Mycoplasma contamination

Cell lines were tested regularly using PanReac AppliChem PCR based Mycoplasma detection kit (Product no. A3744) and were tested negative.

Commonly misidentified lines
(See ICLAC register)

No commonly misidentified cell lines were used

Animals and other organisms

Policy information about studies involving animals; ARRIVE guidelines recommended for reporting animal research

Laboratory animals

Since we used only embryonic samples there is no information on the sex of animals. The embryonic stages of the used embryos are indicated directly in each figure, graph or the figure legends as well as in the main text.
The strain of the mouse lines Syt13F/F, Syt13FD/FD, Ngn3Cre/+; Syt13F/FD, Ins1Cre/+; Syt13F/FD was C57BL/6J and the Syt13F/F; Gt(ROSA)26mTmG, NVF; Syt13FD/FD, and Syt13-Venus fusion lines were on the mixed background (C57BL/6J × 129/SvJ).
The mice were kept at the central facilities at Helmholtz Center Munich (HMGU) under Specific-pathogen-free (SPF) conditions. Animal rooms had a light cycle of 12/12 h, temperature of 20–24 C and humidity of 45–65%. Mice received sterile filtered water and standard diet for rodents ad libitum.
Syt13 gene-trapped (Syt13GT/GT) (EUCOMM) embryonic stem cells were aggregated with CD1 morula to generate chimeric mice. Gene trap mice were bred on a mixed background. For generation of Syt13 full KO mice, first gene trap Syt13 mice were crossed with Flpe mice to obtain floxed mice (Syt13F/F). Then, Syt13 floxed mice were crossed with Rosa26Cre/+ to delete the critical exon 2 and generate the flox deleted (FD) (Syt13FD/FD) mice. Heterozygous (Syt13+/FD) intercross mice were used to obtain full knockout (KO) (Syt13 KO) embryos, which were genotyped by PCR analysis. To generate Syt13 tissue-specific conditional knockout mice, Syt13F/F mice were crossed with constitutive Tg (Neurog3-cre)C1Able/J (Ngn3+/Cre) and Ins1Cre (Ins1tm1(cre)Thor) (Ins1+/Cre) mice.

Wild animals

No wild animals were used in this study.

Field-collected samples

The study did not include field collected samples.

Ethics oversight

Animal studies were conducted with adherence to relevant ethical guidelines for the use of animals in research in agreement with German animal welfare legislation with the approved guidelines of the Society of Laboratory Animals (GV-SOLAS) and the Federation of Laboratory Animal Science Associations (FELASA). The study was approved by the Helmholtz Zentrum München (HMGU) Animal Welfare Body and by the Government of Upper Bavaria.

Note that full information on the approval of the study protocol must also be provided in the manuscript.
